# Metal sulfide ion exchangers: superior sorbents for the capture of toxic and nuclear waste-related metal ions

**DOI:** 10.1039/c6sc01039c

**Published:** 2016-04-26

**Authors:** Manolis J. Manos, Mercouri G. Kanatzidis

**Affiliations:** a Department of Chemistry , University of Ioannina , 45110 Ioannina , Greece; b Department of Chemistry , Northwestern University , Evanston , IL 60208 , USA . Email: m-kanatzidis@northwestern.edu

## Abstract

Metal sulfide ion-exchangers (**MSIEs**) represent a new addition to the field of ion exchange materials. This is a growing class of materials that display exceptional selectivity and rapid sorption kinetics for soft or relatively soft metal ions as a result of their soft basic frameworks. Without requiring functionalization, they outperform the most efficient sulfur-functionalized materials. This is the first review focusing on this class of materials; it covers the most important **MSIEs**, focusing on their synthesis, structural features and ion-exchange chemistry. Furthermore, recent developments in the engineered and composite forms of **MSIEs** are described. Future research opportunities are also discussed in the hope of inspiring additional scientists to engage in this new area of research on sulfidic ion-exchange materials.

## Abstract 2

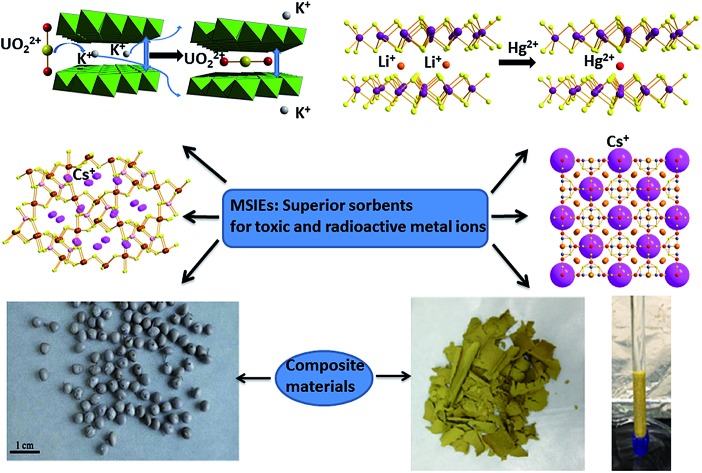
Metal sulfide ion-exchangers (**MSIEs**) have emerged as a new class of promising sorbents for the removal of toxic and radioactive metals from wastewater.

## 1. Introduction

The treatment of various types of aqueous wastes, such as industrial and nuclear waste effluents, is of major concern for countries all over the world. Radionuclides (^137^Cs, ^89^Sr, ^235^U, ^59^Fe, ^57^Co, ^65^Zn, *etc.*) and toxic heavy metal ions (Hg^2+^, Pb^2+^, Cd^2+^, and Tl^+^) are major pollutants in these types of waste and pose a serious threat to humans and other species.^1^ Commonly used and inexpensive methods such as precipitation of the ions from solutions are often not sufficiently effective to lower the concentration of these ions below the acceptable legal limits. For example, precipitation of Hg^2+^ ions with Na_2_S cannot reduce the concentration of mercuric ions below 10 to 50 ppb.^2^ Such levels are 20 to 100-times higher than the legally accepted limits defined by the European Union (1 ppb) and USA-EPA (2 ppb).^3^ The issue of the treatment of nuclear effluents is even more complex because of the harsh conditions of nuclear waste deriving from nuclear waste manufacture, such as inhomogeneous samples, extreme pH and very high salt concentrations.^4^ Efficient removal of radioactive elements and minimization of long-term storage space is crucial to enable safer and low cost implementation of nuclear energy.^4^

Ion exchange is well recognized as a relatively inexpensive and highly effective method for the elimination of various types of ions from aqueous waste solutions.^5^ Clays^6^ and zeolites^7^ are common and abundant cation exchangers; however, they suffer from low selectivity and capacity for toxic heavy metal ions in the presence of high salt concentrations or under acidic conditions. In addition, these materials are unstable in extreme alkaline or acidic conditions (due to immediate dissolution of aluminum/silicon ions) of nuclear wastes.^8^ Other oxidic sorbents, such as titanates, silicates and manganese oxides, can survive under the conditions of nuclear waste; however, they show decreased selectivity for radioactive ions in the presence of high salt concentrations (*e.g.* Cs^+^ absorption by manganese oxides),^9^ or they are selective for radioactive ions only within a narrow pH range (*e.g.* Sr^2+^ absorption by sodium titanate).^10^

Organic resins, with functional groups suitable for absorption of specific ions, are extensively used for water purification.^5^ These purely organic materials, however, are of limited chemical, radiolytic and thermal stability.^8^ In addition, resins display an amorphous porous structure; therefore, they cannot exhibit the molecular sieve separation properties of ordered porous inorganic materials such as zeolites.^5^

Functionalized silica-based materials show remarkable selectivity and binding affinity for a variety of heavy ions. For example, thiol-functionalized mesoporous materials are famous for their exceptional capability to rapidly absorb Hg^2+^ from water solutions.^11^ Silica-based materials, however, cannot be used for remediation of extreme alkaline or acidic waste water (*e.g.* nuclear waste) due to their instability under such conditions.^8^

Metal organic frameworks (MOFs) incorporating functional groups with high affinity for toxic or radioactive ions appear to be promising sorbents for various remediation processes.^12^ The development of these sorbents is still in its infancy.

From the above, it is clear that “perfect” sorbents that can withstand the harsh conditions of various types of wastes, are highly selective for toxic or radioactive ions, and are affordable are still elusive. The search for new sorbent materials is therefore important.

Recently, metal sulfide ion exchangers (**MSIEs**) with labile extra-framework cations have emerged as a new class of promising sorbents.^13^ These materials exhibit a variety of structures, ranging from layered and three-dimensional crystalline frameworks^13^ to porous amorphous materials^14^ and aerogels.^15^ They are proving to be particularly effective for the decontamination of water solutions from various heavy metal ions (*e.g.* Hg^2+^, Pb^2+^, Cd^2+^, Ni^2+^, and Co^2+^) as well as ions relevant to nuclear waste (*e.g.* UO_2_^2+^, Cs^+^, and Sr^2+^).^13^ The unique properties of **MSIEs** arise from their soft S^2–^ ligands, which endow these materials with innate selectivity for soft or relatively soft metal ions. **MSIEs** with a soft basic framework thus do not require the introduction of any functional groups. They exhibit exceptional absorption properties for soft metal ions, superior to those of the best sulfur-functionalized materials.^11^ Furthermore, hard ions such as H^+^, Na^+^, and Ca^2+^ only weakly interact with the soft S^2–^ ligands of **MSIEs**, thus affecting their ion exchange properties to a much lesser degree than those of traditional oxidic materials.^6^,^7^,^9^,^10^ Therefore, **MSIEs** may be effective for metal ion absorption over a broad pH range and in the presence of high salt concentration. In this review, we describe the most important **MSIEs** in terms of their synthesis, structural characteristics and metal ion absorption properties. Furthermore, we discuss the recent development of composite and engineered forms of **MSIEs**, which promise to open up paths for the practical applications of these materials. To our knowledge, this is the first review of these systems. Finally, we provide some directions and perspectives for future research on this new family of ion-exchangers.

## 2. Layered crystalline MSIEs

Metal sulfides with layered anionic structures and labile interlayer cations constitute the most well studied class of **MSIEs**. These materials show excellent and selective ion exchange properties due to (a) the facile diffusion of the inserted ions and their easy access to the internal surface of metal sulfide layers and (b) the formation of strong bonds between the incorporated metal ions and S^2–^ ligands. In the following, we present the main layered **MSIEs**, highlighting their synthesis, structural features and ion exchange chemistry.

### 2.1. Alkali-ion intercalated metal disulfides

A common route to the preparation of layered **MSIEs** involves partial reduction of the metal ions of a metal disulfide, such as SnS_2_ or MoS_2_, by treating it with an alkali ion (A^+^)-containing reducing agent (*e.g. n*-butyl lithium, alkali ion dithionite).^16^ An anionic layer is thus formed, and its negative charge is compensated by alkali cations provided by the reducing agent. The cations inserted in the interlayer space of the materials can be rapidly and topotactically exchanged by a variety of inorganic and organic cationic species.^16^ The synthesis of such layered materials is represented by the following equation:

1 MS_2_ + *x*A^+^ + *x*e^–^ → A_*x*_[MS_2_]^*x*–^

Actually, the earliest known **MSIEs**, which were reported on 1979 by R. Schöllhorn *et al.*, were alkali-ion intercalated metal disulfides, specifically the hydrated layered Sn sulfide phases A_*x*_(H_2_O)_*y*_[SnS_2_]^*x*–^ (A^+^ = K^+^, Rb^+^, Cs^+^).^17^ These materials show facile ion-exchange properties for a series of inorganic cations, such as Li^+^, Na^+^, Mg^2+^, Ca^2+^, and Ni^2+^. The insertion of the various ions in the interlayer space of the materials was probed by powder X-ray diffraction (PXRD) studies. It was observed that the higher the hydration energy of the inserted cation, the larger the interlayer spacings, indicating the incorporation of the ions as hydrated complexes in the space between the layers. These materials, however, are susceptible to oxidation, which results in SnS_2_.

**Fig. 1:**
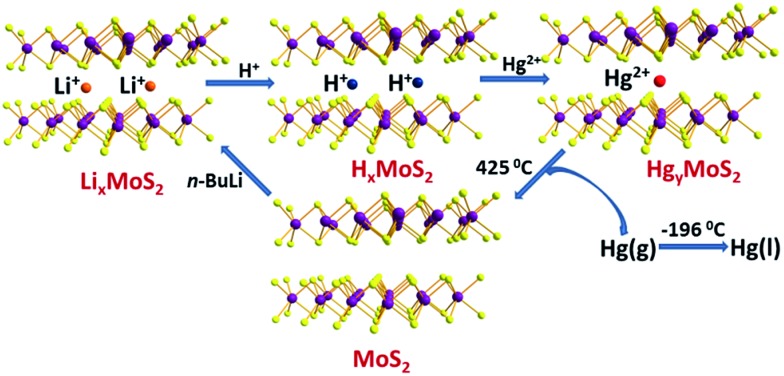
Suggested mechanism of Hg^2+^ absorption/desorption for the Li_*x*_MoS_2_ material. Mo, purple; S, yellow.

About twenty years after Schöllhorn, the ion-exchange properties of Li_*x*_MoS_2_ (0.25 ≤ *x* ≤ 1.9) were investigated by A. E. Gash *et al.*^18^ This material is particularly effective in binding to Hg^2+^ in acidic conditions. The authors suggested a mechanism for the Hg^2+^ absorption, which is shown in Fig. 1 and represented by the following equations:

2 Li_1.3_MoS_2_(s) + 1.3H_3_O^+^ → 0.26H_2_(g) + 1.3Li^+^(aq) + (H_3_O)_0.78_MoS_2_(s) + 0.52H_2_O(l)

3 (H_3_O)_0.78_MoS_2_(s) + 0.20Hg^2+^(aq) → (H_3_O)_0.38_Hg_0.20_MoS_2_(s) + 0.40H_3_O^+^(aq)

Thus, the first step of the ion-exchange reaction involved the transformation of the Li^+^-intercalated material to a hydronium-containing material, which in turn reacts with Hg^2+^ to yield the final product. The driving force for the second reaction was the higher affinity of the soft basic MoS_2_^*x*–^ layers for the soft acid Hg^2+^ compared to that for the hard hydronium ions. Inductively coupled plasma-atomic emission (ICP-AES) analytical data indicated that ∼200 ppm of Hg^2+^ (pH ∼ 1) can be reduced to 6.5 ppb after treatment of the Hg^2+^ solution with Li_*x*_MoS_2_ (the molar ratio of Li_*x*_MoS_2_ to Hg was 5). The Hg content of various exchanged products was found to be 0.24 to 0.32 mole per formula unit of the exchanged material, depending on the initial Li_*x*_MoS_2_/Hg molar ratio.

Interestingly, Hg can be recovered by heating the Hg-exchanged product at 425 °C, which leads to the vaporization of Hg and the formation of MoS_2_ (Fig. 1) according to the following equation:

4 Hg_*x*_MoS_2_(s) → *x*Hg(g) + MoS_2_(s)

The Hg vapor is then collected in a cold trap at 77 K. The MoS_2_ can be retransformed to Li_*x*_MoS_2_*via* reduction-Li intercalation with *n*-BuLi. As observed for the alkali-intercalated tin disulfide materials, Li_*x*_MoS_2_ sorbents are also air and moisture-sensitive and thus, they should be stored under anaerobic conditions to retain their Hg^2+^ sorption capacity.

Li_*x*_MoS_2_ was also tested for Zn^2+^, Pb^2+^ and Cd^2+^ sorption.^18^ The results revealed only moderate Pb^2+^ removal capacity (40–75% removal of the initial Pb content), relatively low Cd^2+^ (4–40% removal capacity) and negligible Zn^2+^ sorption (1–4% removal capacity). In contrast, the removal capacities for Hg^2+^ were 74–100%. The selectivity of the Li_*x*_MoS_2_ follows the order Hg^2+^ > Pb^2+^ > Cd^2+^ > Zn^2+^, which indicates the preference of the material for softer metal ions due to their stronger interactions with the soft MoS_2_^*x*–^ layer.

### 2.2. KMS materials

**Fig. 2:**
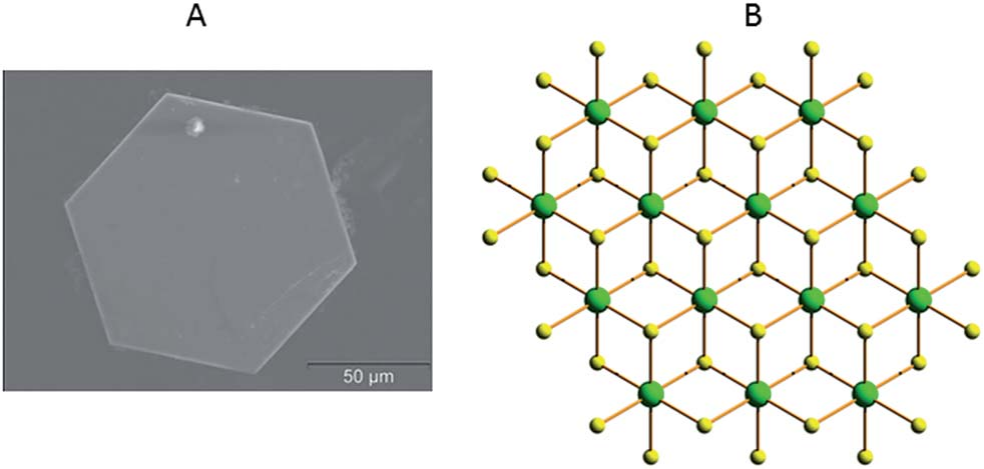
(A) Scanning electron microscopy (SEM) image of a typical crystal of KMS-1. (B) Representation of part of the layered structure of KMS-1 (S, yellow; Sn/Mn, green).

**Fig. 3:**
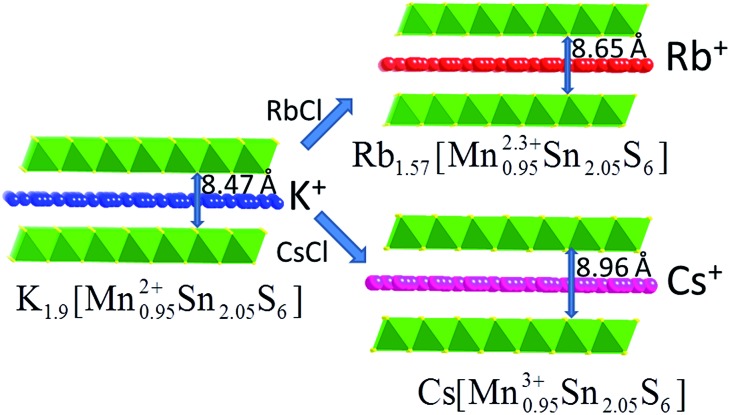
Representations of the crystal structures of KMS-1 and its Rb^+^ and Cs^+^-exchanged analogues and the interlayer spacing.

These materials have the general formula K_2*x*_M_*x*_Sn_3–*x*_S_6_ (M = Mn^2+^, KMS-1; M = Mg^2+^, KMS-2; *x* = 0.5 to 1).13c–h They can be prepared on a multigram scale with high purity using solid state and hydrothermal synthetic methods. They are exceptionally stable in air and in highly acidic and basic aqueous solutions. Single crystal X-ray measurements, performed on hexagonal-shaped crystals of KMS-1 and 2 (Fig. 2A), revealed that their structure is based on edge sharing “Sn/M”S_6_ octahedra (CdI_2_ structure type) with Sn^4+^ and M^2+^ occupying the same crystallographic position and three-coordinated S^2–^ ligands (Fig. 2B). The interlayer space is filled by K^+^ ions (Fig. 3), which compensate for the negative charge of the metal sulfide layers. There is much more room in the interlayer space than that required for all K^+^ cations. As a result, these ions are highly disordered and mobile and are thus easily exchanged by a variety of other cationic species (see below). KMS compounds are actually derivatives of SnS_2_ with partial substitution of Sn^4+^ by M^2+^ (Mn^2+^ or Mg^2+^) ions. KMS-1 and 2 feature essentially the same structural characteristics; their only differences are related to the stacking of the layers.

KMS-1 and 2 materials were investigated in detail for their ion-exchange properties with cations related to nuclear waste, such as Cs^+^, Sr^2+^, Ni^2+^ and UO_2_^2+^, and heavy metal ions (such as Hg^2+^, Pb^2+^, Cd^2+^) which are common contaminants in industrial wastewater. In the following, we describe the most important ion-exchange results for KMS materials.

#### 2.2.1. Cs^+^ and Rb^+^ ion exchange properties

The Cs^+^ ion-exchange process is highly relevant to nuclear waste remediation, since the ^137^Cs^+^ radionuclide represents one of the major contaminants in the fission products of nuclear waste.^4^ It is thus of particular importance to discover selective Cs^+^ sorbents and expand the gamut of materials that can be used to capture this ion. For this reason, the Cs^+^ ion-exchange properties of KMS-1 and 2 were investigated in detail. For comparison, Rb^+^ absorption was also studied.

K^+^ ions can be completely and topotactically replaced by Cs^+^ or Rb^+^ by treating KMS-1 with an aqueous solution of the corresponding alkali-ion chloride salt. Interestingly, Cs^+^ and Rb^+^ sorption can be achieved not only with polycrystalline samples of KMS-1, but also in a single crystal-to-single crystal (**SCSC**) fashion using large specimens.13*d* When single crystals of K_2*x*_Mn_*x*_Sn_3*x*_S_6_ (*x* = 0.95, KMS-1) are immersed in a solution of CsCl or RbCl, single crystals of Cs^+^ or Rb^+^-exchanged material can be isolated and, thus, their structures can be accurately determined by X-ray crystallography (Fig. 3). X-ray refinement indicated the formulas Rb_1.57_Mn_0.95_Sn_2.05_S_6_ and CsMn_0.95_Sn_2.05_S_6_ for the Rb^+^ and Cs^+^-exchanged materials, respectively. Based on these formulas and the charge balance requirements, Mn should be in the 2.3+ and 3+ oxidation states in the Rb^+^ and Cs^+^-containing compounds, respectively. The Mn oxidation states in these exchanged materials were also confirmed using X-ray photoelectron spectroscopic (XPS) data. The stability of the Mn^3+^ oxidation state in the Cs^+^-exchanged analogue was explained on the basis of the increased ionic character of the Cs^+^···S^2–^ interactions. Thus, in the presence of Cs^+^, the [Mn_*x*_Sn_3–*x*_S_6_]^2*x*–^ layer becomes more electron-rich and, as a consequence, becomes more prone to lose an electron.13*d*

Detailed batch Cs^+^ ion exchange studies were performed for KMS-1, and the data can be described well with the Langmuir model:
5

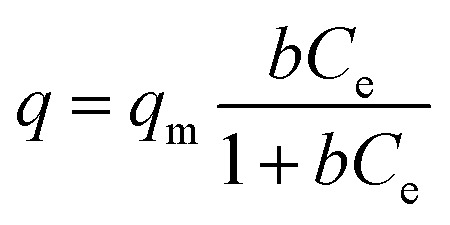

where *q* (mg g^–1^) is the amount of the cation absorbed at the equilibrium concentration *C*_e_ (ppm), *q*_m_ is the maximum sorption (exchange) capacity of the sorbent, and *b* (L mg^–1^) is the Langmuir constant related to the free energy of the sorption.13*d*

**Fig. 4:**
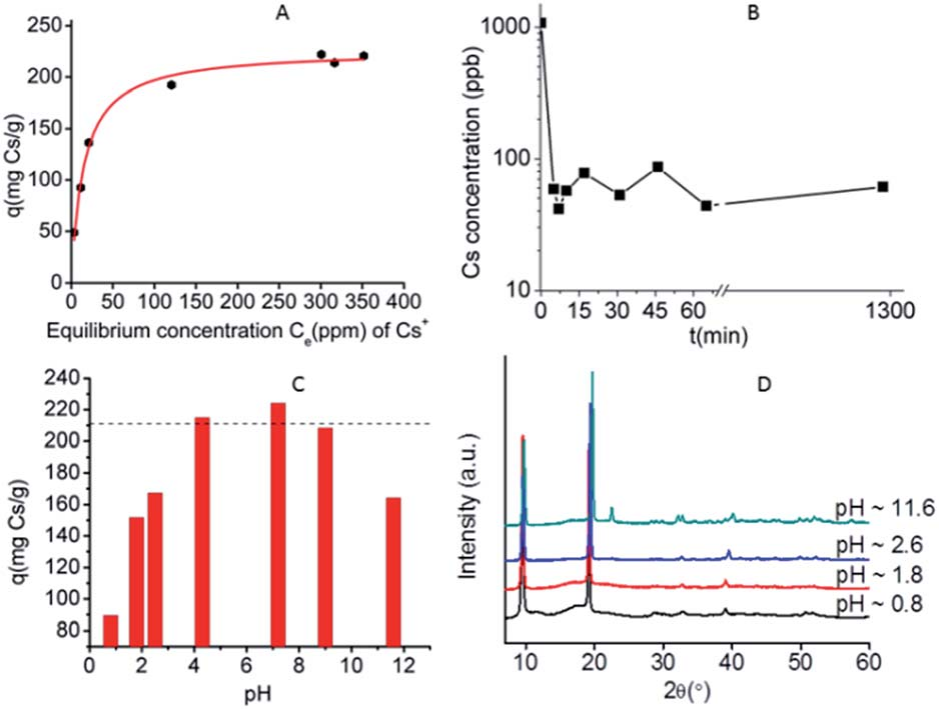
(A) Cs^+^ isotherm sorption data for KMS-1 (at pH ∼ 7). The solid line represents the fitting of the data with the Langmuir model. (B) Kinetic data for the sorption of Cs^+^ (initial concentration ∼ 1 ppm) by KMS-1. (C) Maximum Cs^+^ sorption capacity of KMS-1 found for different pH values. The dashed line represents the maximum theoretical exchange capacity for KMS-1. (D) PXRD patterns for Cs^+^-exchanged products isolated from highly acidic and alkaline solutions.

The fitting of the data indicated a maximum sorption capacity of ∼1.7 mmol g^–1^ (226 ± 2 mg g^–1^), close to the maximum theoretical capacity (1.6 mmol g^–1^) calculated for the exchange of two K^+^ ions by one Cs^+^ (Fig. 4A). The material can be regenerated by treating the Cs^+^-loaded compound with a large excess of KCl aqueous solution. The regenerated compound showed almost identical Cs^+^ exchange capacity to that of the pristine KMS-1 material.

Kinetic studies with trace Cs^+^ concentrations (∼1 ppm) revealed a very fast sorption process at room temperature. Specifically, within less than 5 min of contact with the KMS-1/solution, the Cs^+^ ion exchange reached its equilibrium with ∼90% removal of the initial Cs^+^ content (Fig. 4B). The material was also capable of efficient Cs^+^ ion-exchange in highly acidic (pH ∼ 1) and alkaline conditions (pH ∼ 12), as shown in Fig. 4C. PXRD studies indicate that the Cs^+^-exchanged products isolated from either acidic or alkaline solutions were crystalline and retained the layered structure of the parent KMS-1 compound (Fig. 4D).

Finally, ion-exchange studies have been performed with solutions simulating alkaline groundwater contaminated by radioactive elements. These solutions with pH ∼ 11 contained low Cs^+^ levels (∼1 ppm) and relatively high concentrations (7 to 125 ppm) of Na^+^, Mg^2+^ and Ca^2+^. Despite the presence of various competitive ions, KMS-1 showed high removal capacities (74–99%) for Cs^+^ from these complex solutions.

KMS-2 also exhibits Cs^+^ ion-exchange properties.13*h* The maximum sorption capacity of KMS-2 is 531 ± 28 mg g^–1^. This is 2.35 ± 0.12 times the capacity of KMS-1 and one of the highest capacities ever reported for Cs^+^ ion-exchangers.^10^ KMS-2 is expected to exhibit twice the Cs^+^ exchange capacity of KMS-1 because in the latter, the sorption capacity is reduced by the oxidation of Mn^2+^ to Mn^3+^ (see above). This oxidation cannot occur in KMS-2 containing Mg^2+^; thus, two equivalents of Cs^+^ can be absorbed by one mole of KMS-2. Furthermore, KMS-2 shows high Cs^+^ sorption capacity not only under neutral pH conditions, but also in acidic (pH = 3) and alkaline (pH = 10) environments.

#### 2.2.2. Sr^2+^ ion exchange properties

Sr^2+^ ion-exchange and capture is also an important process, since radioactive ^90^Sr^+^ represents one of the major heat producers and biohazards in nuclear waste.^4^ Thus, detailed Sr^2+^ ion-exchange property studies were performed with KMS-1 and 2.13c,h The isotherm sorption data for these materials can be fitted with the Langmuir model and revealed maximum Sr^2+^ sorption capacities of 77 ± 2 and 87 ± 2 mg g^–1^ for KMS-1 and 2, respectively. Both KMS-1 and KMS-2 perform very efficiently in a very broad pH range. The affinity of the sorbents for Sr^2+^ was expressed in terms of the distribution coefficient *K*_d_, which is calculated by the equation:
6

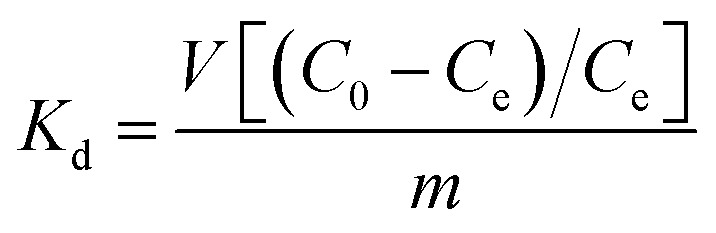

where *C*_0_ and *C*_e_ are the initial and equilibrium concentrations of Sr^2+^ (ppm), respectively, *V* is the volume (mL) of the testing solution and *m* is the amount of the ion exchanger (g) used in the experiment.^13^ Values of *K*_d_ equal to or above 10^4^ mL g^–1^ are considered excellent. KMS-1 showed very high *K*_d_ values in the range of 10^4^ to 10^5^ mL g^–1^ from pH 3 to 14.

KMS-1 performs very efficiently for Sr^2+^ sorption. Its removal capacity is ∼92% (*K*_d_ ∼1.2 × 10^4^ mL g^–1^) at very high salt concentrations (∼5 M Na^+^) and pH ∼ 14, which are the conditions typically found in alkaline nuclear waste. KMS-1 outperforms oxidic sorbents for Sr^2+^ ion exchange under acidic conditions. The KMS-1 material contains soft basic S^2–^ ligands with very small affinity for protons. This is not the case for the conventional oxidic exchangers, where the hard H^+^ ions show great affinity for O^2–^ ligands, which interferes with the process.^19^–^21^

**Fig. 5:**
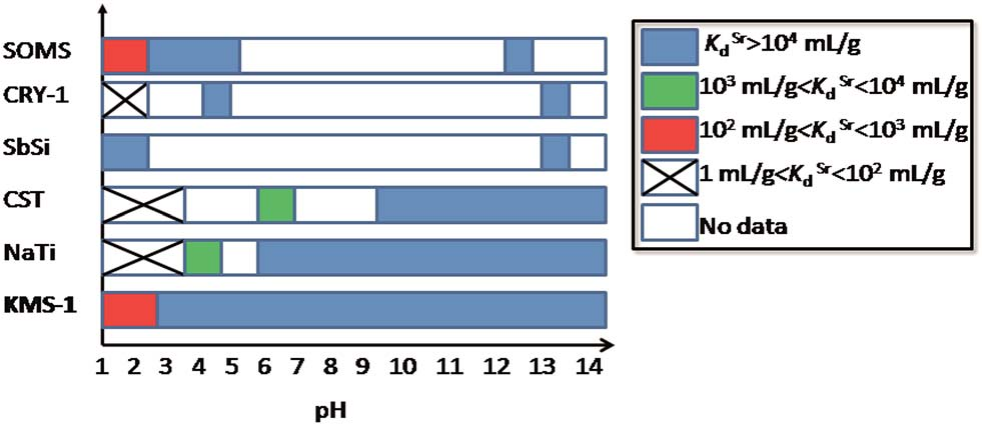
Comparison diagram indicating the dependence of *K*_d_ on pH for the Sr^2+^ ion-exchange of various materials. NaTi: sodium titanate,^19^ CST: sodium silicotitanate,19b,d CRY-1: cryptomelane-type manganese oxide,^20^ SOMS: Sandia octahedral molecular sieves^21^ and SbSi: Sb silicate.19*b*

The comparison of KMS-1 with various Sr^2+^ sorbents, in terms of their *K*_d_ values *vs.* the pH of the solution, is provided in Fig. 5.

KMS-2 is also efficient in removing Sr^2+^ under both alkaline and acidic conditions. Thus, *K*_d_ values of 6.3 × 10^4^ and 1.5 × 10^5^ mL g^–1^ were observed for the Sr^2+^ exchange of KMS-2 at pH ∼ 3 and 10, respectively.

#### 2.2.3. Ni^2+^ ion-exchange properties

**Fig. 6:**
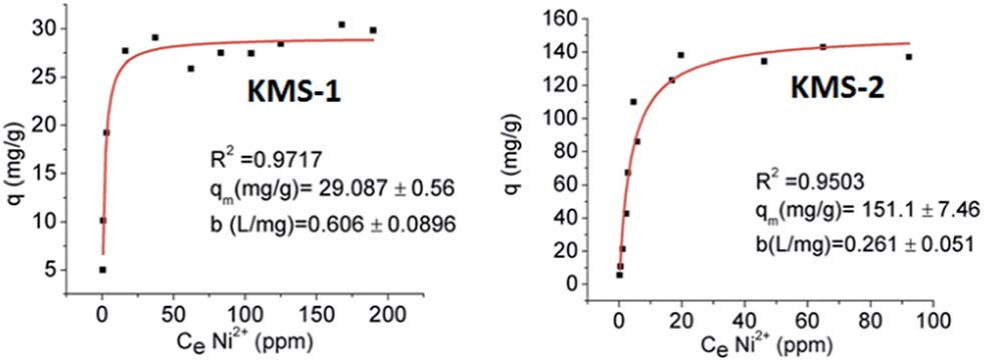
Ni^2+^ isotherm sorption data for KMS-1 and 2. The solid lines represent the fitting of the data with the Langmuir model.

During the nuclear fission process, radioactive byproducts are generated due to the corrosion of the containers by the heat and acidity produced. Among these byproducts, ^63^Ni is regulated as a low energy beta contaminant.^4^ Thus, the removal of Ni^2+^ from aqueous solutions is relevant to the decontamination of nuclear waste from the corrosion products of the fission process. For this reason, the Ni^2+^ ion-exchange properties of KMS-1 and 2 were investigated (Fig. 6).13*h*

The isotherm Ni^2+^ sorption data for KMS-1 and 2 follow the Langmuir model and indicate maximum sorption capacities of ∼29 and 151 mg g^–1^, respectively. The relatively low Ni^2+^ sorption capacity of KMS-1 is likely due to the oxidation of Mn^2+^ to Mn^3+^. In the case of KMS-2, the observed Ni^2+^ sorption capacity is ∼1.4 larger than the theoretically expected value. This is attributed to the exchange not only of the interlayer K^+^ ions but also the intralayer Mg^2+^ ions. KMS materials are highly selective for Ni^2+^ in the presence of a tremendous excess of Na^+^ ions. Thus, a reasonably high *K*_d_ value of 1.8 × 10^5^ mL g^–1^ was obtained for Ni^2+^ exchange of KMS-2 (initial Ni^2+^ concentration of ∼6 ppm) in the presence of 5 M Na^+^. The hard Na^+^ ions exhibit very weak interactions with the soft metal sulfide layer, in contrast to the relatively soft Ni^2+^, which may interact strongly with the soft S^2–^ ligands. As a result, high Na^+^ concentrations have a negligible effect on the Ni^2+^ exchange of KMS materials. This impressive binding to Ni^2+^ ions also indicates that KMS materials may be effective agents in Ni recovery from industrial effluents arising from massive nickel plating operations worldwide.^22^

#### 2.2.4. Hg^2+^, Pb^2+^ and Cd^2+^ ion-exchange and capture

**Fig. 7:**
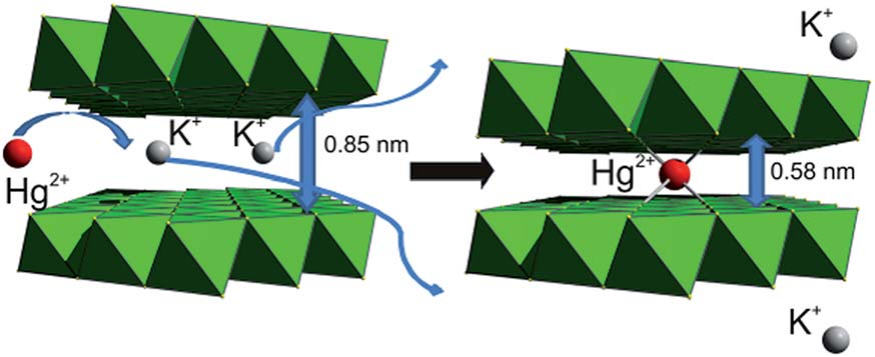
Capture of Hg^2+^ by KMS-1 through exchange of its interlayer potassium cations.

Because Hg^2+^, Pb^2+^ and Cd^2+^ ions present a major health hazard for drinking and industrial wastewater,^1^ it is important to develop highly selective sorbents for these ions with very high loading capacities.^11^ The layered structure of KMS-1 sorbent and the presence of soft basic sites (S^2–^ ligands) allow for very rapid kinetics for the exchange of interlayer K^+^ ions by soft Lewis acids, such as Hg^2+^, Pb^2+^ and Cd^2+^ ions (Fig. 7).13*e*

PXRD studies indicated that significant contraction of the interlayer space occurs upon exchange of K^+^ ions by Hg^2+^ (Fig. 7), which is due to the smaller size of the Hg^2+^ ion compared to K^+^ and the formation of strong Hg^2+^–S^2–^ covalent interactions.

The PXRD pattern for the Pb^2+^ exchanged product showed the presence of two interlayer spacings because of the different hydrations of the Pb^2+^ ions ([Pb(H_2_O)_*x*_]^2+^). The Cd^2+^ ion-exchange process is particularly interesting. The analytical data for the Cd^2+^-exchanged product showed the absence not only of K^+^ ions but also of Mn^2+^. Specifically, the average formula of the Cd^2+^-loaded compound was determined as Cd_1.8_Sn_2.1_S_6_, and the ion-exchange process is described by the following equation:

7 K_1.9_Mn_0.95_Sn_2.05_S_6_ + 1.9Cd^2+^ → [Cd(H_2_O)_1.5_]_0.95_[Cd_0.95_Sn_2.05_S_6_] + 0.95Mn^2+^ + 1.9K^+^

**Fig. 8:**
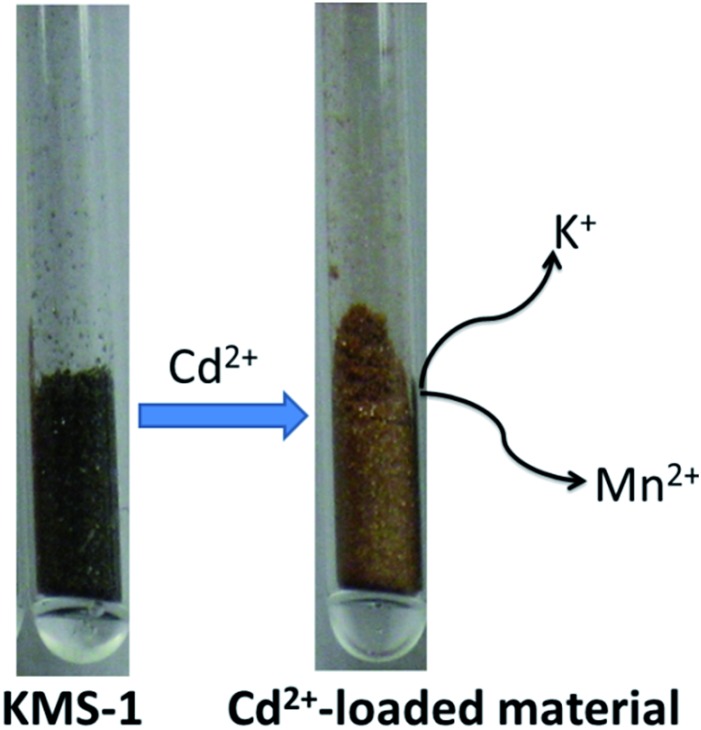
Color change of KMS-1 upon the Cd^2+^ exchange process.

The Cd^2+^-exchanged KMS-1 has a layered structure, as indicated by the PXRD data, and the Cd^2+^ is inserted as a hydrated ion in the interlayer space and also replaces all intra-layer Mn^2+^ ions. Due to the complete exchange of Mn^2+^ by Cd^2+^, a dramatic color change of the material from dark brown to orange-brown was observed upon Cd^2+^ exchange (Fig. 8).

Isotherm batch sorption data revealed maximum sorption capacities of 320 to 377 mg g^–1^ for the Hg^2+^, Pb^2+^ and Cd^2+^ exchange processes. The capture of these ions by KMS-1 is not affected by the acidity or basicity of aqueous solutions. Thus, very high *K*_d_ values (>10^4^ mL g^–1^) for these ions in a broad pH range (2.5 to 10) can be achieved. In addition, the presence of a very large excess of Na^+^ and Ca^2+^ ions, which are commonly found in high concentrations in drinking water and industrial wastewater, has no effect on the exchange of Hg^2+^ and Pb^2+^ and only slightly interferes with the Cd^2+^ exchange process.

**Fig. 9:**
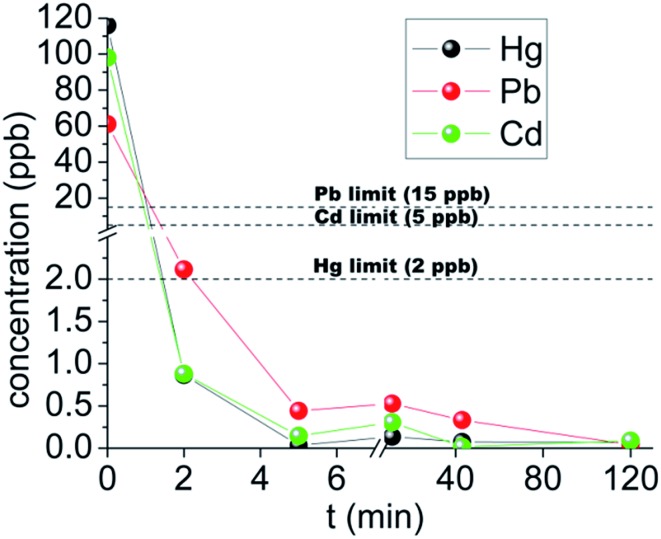
The kinetics for the decontamination of potable water sample intentionally contaminated by mercury (116 ppb), lead (61 ppb) and cadmium (98 ppb) with KMS-1.

When Hg^2+^, Pb^2+^ and Cd^2+^ are present simultaneously in solution, KMS-1 can capture all three ions with no apparent selectivity. The concentrations of all three ions are reduced well below the safety limits within only 2 min of KMS-1/solution contact (Fig. 9). KMS-2 also exhibits exceptional Hg^2+^, Pb^2+^ and Cd^2+^ exchange properties.13*e* This result is in marked contrast to thiol-functionalized materials that show selectivity for Hg^2+^ and exhibit low to moderate sorption capacity for Pb^2+^ and Cd^2+^.^11^

Regeneration of KMS-1/2 from the Hg^2+^, Pb^2+^ or Cd^2+^-loaded materials was not feasible because of the very strong binding of these ions. Because of the extremely high metal ion sorption capacity (up to 50% by weight) and relatively low cost of KMS materials, it may not be necessary to regenerate them; thus, the ion-loaded materials could be considered as a permanent waste form, suitable for safe disposal. Initial investigations indicate negligible leaching of Hg^2+^, Pb^2+^ or Cd^2+^ from the corresponding exchange products after their hydrothermal treatment at 70 °C for 24 h.

Finally, a methylammonium analogue of KMS-1, with the formula [CH_3_NH_3_]_2*x*_Mn_*x*_Sn_3–*x*_S_6_·0.5H_2_O (*x* = 1.0 to 1.1) (CMS),^23^ was recently described, and its Cd^2+^ and Pb^2+^ ion-exchange properties were studied. The results of these investigations revealed rapid sorption kinetics, high sorption capacity and exceptional selectivity of CMS for Cd^2+^ and Pb^2+^ (see also the section below for a comparison between MS exchangers, and Table 1).

**Table 1:** Important ion-exchange characteristics of representative **MSIEs**

Material	*q*_m_ ^*a*^ (mg g^–1^)	*K*_d_ (mL g^–1^) individual (pH ∼ 7)	*K*_d_ (mL g^–1^) competitive (Na^+^)	*K*_d_ (mL g^–1^) competitive (Ca^2+^)	Active pH range	Kinetic studies-equilibrium time (min) at RT	Reference
**Cs** ^**+**^							
KMS-1	226 ± 2	≥10^4^	≥10^3^ ^*e*^	≥10^2^ ^*f*^	0.8–12	5	13*d*
KMS-2	531 ± 28	≥10^3^	≥10^*g*^	NA^*b*^	3–10	NA	13*h*
KTS-1	205 ± 6	≥10^5^	NA	NA	NA	NA	33
KTS-3	280 ± 11	≥10^4^	≥10^3^ ^*h*^	NA	2–12	5	13*i*
FJSM-SnS	409 ± 29 (65 °C)	≥10^3^	NA	NA	0.7–11	5 (65 °C), 30 (RT)	34
GeSbS-2	231 ± 15 (65 °C)	≥10^3^	NA	NA	2.8–11	2 (65 °C)	39
K_6_MS	66 ± 4	≥10^4^	≥10^4^ ^*i*^	≥10^3^ ^*j*^	2.5–12	NA	42

**Sr** ^**2+**^							
KMS-1	77 ± 2	≥10^5^	≥10^5^ ^*k*^	NA	1–14	NA	13*c*
KMS-2	87 ± 2	≥10^4^	≥10^*l*^	NA	3–10	NA	13*h*
KTS-3	102 ± 5	≥10^5^	≥10^2^ ^*m*^	NA	2–12	5	13*i*
FJSM-SnS	65 ± 5	≥10^4^	NA	NA	0.7–11	5 (65 °C), 60 (RT)	34

**Hg** ^**2+**^							
KMS-1	377 ± 15^*c*^	≥10^5^	≥10^5^ ^*n*^	≥9 × 10^4^ ^*n*^	2.5–9.5	5	13*e*
LHMS-1	87 ± 6^*c*^	≥10^6^	≥10^5^ ^*n*^	≥10^5^ ^*n*^	0–9	10	13*f*
K_6_MS	NA	≥10^6^	≥10^6^ ^*n*^	≥10^6^ ^*n*^	3–8	60	43

**Pb** ^**2+**^							
KMS-1	319 ± 4^*c*^	≥10^6^	≥8 × 10^4^ ^*n*^	≥10^4^ ^*n*^	2.5–9.5	5	13*e*
CMS	1053^*d*^	≥10^6^	≥10^6^ ^*n*^	≥10^6^ ^*n*^	1–7	30	23

**Cd** ^**2+**^							
KMS-1	329^*d*^	≥10^7^	≥6 × 10^3^ ^*n*^	≥7 × 10^3^ ^*n*^	0–9	5	13*e*
CMS	515^*d*^	≥10^5^	≥10^5^ ^*n*^	≥10^5^ ^*n*^	1–7	90	23

**UO**_**2**_^**2+**^							
KMS-1	380 ± 20	≥10^5^	≥10^5^ ^*o*^	≥10^4^ ^*p*^	2.5–10	120^*q*^	13*g*
KTS-3	287 ± 15	≥10^4^	≥5 × 10^3^ ^*o*^	NA	2–12	50^*r*^	13*i*

**Ni** ^**2+**^							
KMS-1	29 ± 1	≥10^5^	≥10^5^ ^*s*^	NA	3–10	NA	13h
KMS-2	151 ± 8	≥10^5^	≥10^5^ ^*s*^	NA	3–10	NA	13h

**Cu** ^**2+**^							
KMS-1	155	≥10^5^	≥10^4^ ^*t*^	≥10^4^ ^*t*^	2–6	20	29

**Tl** ^**+**^							
K_6_MS	508 ± 31^*c*^	≥10^5^	NA	NA	NA	NA	43

^*a*^Estimated from the fitting of the data with the Langmuir model.

^*b*^Not available.

^*c*^Estimated from the fitting of the data with the Langmuir–Freundlich model.

^*d*^Estimated by averaging the metal uptake values corresponding to the saturation of the exchange sites of the material.

^*e*^Na : Cs molar ratio ∼655, pH ∼ 7.

^*f*^Ca : Cs molar ratio ∼21.

^*g*^Na : Cs molar ratio ∼1.1 × 10^5^, pH ∼ 7.

^*h*^Na : Cs molar ratio ∼1.8 × 10^3^.

^*i*^Na : Cs molar ratio ∼1.3 to 6.5 × 10^5^.

^*j*^Ca : Cs molar ratio ∼10^5^.

^*k*^Na : Sr molar ratio ∼1887.

^*l*^Na : Sr molar ratio ∼1.9 × 10^5^.

^*m*^Na : Sr molar ratio ∼329.

^*n*^Competitive ion: Hg^2+^ (or Pb^2+^ or Cd^2+^) molar ratio ≥ 1000.

^*o*^Na : U molar ratio ≥ 10^4^.

^*p*^Ca : U molar ratio ≥ 10^4^.

^*q*^97% of U removal is achieved within 2 min.

^*r*^80% removal is achieved within 5 min.

^*s*^Na : Ni molar ratio ∼4.8 × 10^4^.

^*t*^Na or Ca/Cu molar ratio ∼8.

#### 2.2.5. Extraction of Ag^+^ and Hg^2+^ ions from their cyanide complexes

Cyanide ions are widely used for the dissolution of precious metals, such as gold and silver, from their minerals. In addition to the precious metal ions, the minerals also contain several heavy metal ions that must be removed and separated from the precious metals.^24^ One example is the mineral Ag_2_Hg_2_S_2_. The cyanidation of this mineral results in the formation of [Hg(CN)_4_]^2–^ and [Ag(CN)_2_]^–^ complexes.^25^ Remarkably, KMS materials can extract Hg^2+^ and Ag^+^ from their cyanide complexes. KMS-2 was studied for these ion-exchange processes.^26^ A complete exchange of the K^+^ ions of KMS-2 by Hg^2+^ or Ag^+^ was observed after treatment of KMS-2 with aqueous solutions of [Hg(CN)_4_]^2–^ or [Ag(CN)_2_]^–^. KMS-2 was also capable of simultaneously and quantitatively capturing Hg^2+^ and Ag^+^ from water solutions containing both [Hg(CN)_4_]^2–^ and [Ag(CN)_2_]^–^ complexes (removal capacities >97%).

**Fig. 10:**
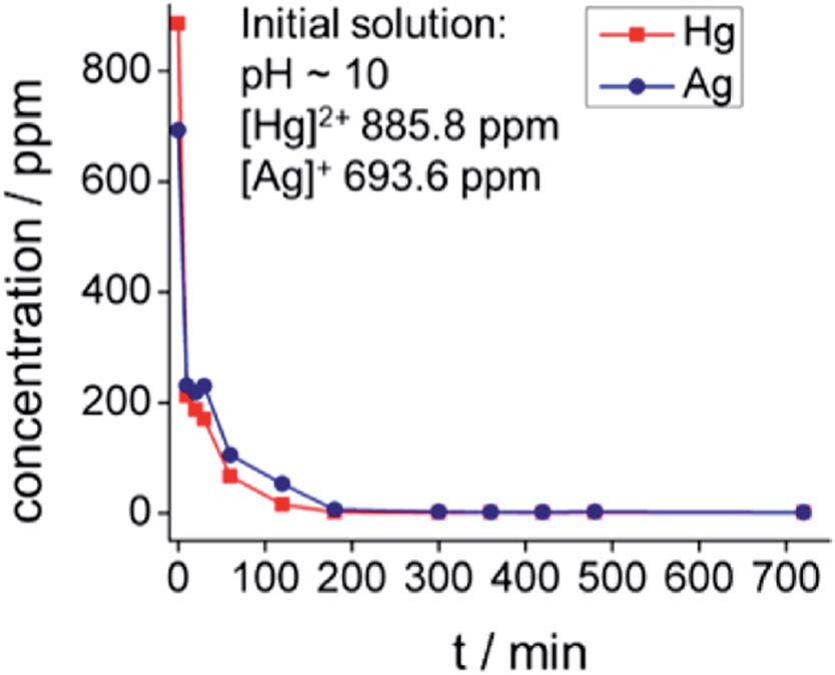
Kinetic data for the sorption of Hg^2+^ and Ag^+^ from a mixture of Hg(CN)_4_]^2–^–[Ag(CN)_2_]^–^ complexes in solution (pH ∼ 10). Reproduced with permission from ref. 26. © 2015, American Chemical Society.

The kinetics of the sorption of Hg^2+^ and Ag^+^ from mixed [Hg(CN)_4_]^2–^–[Ag(CN)_2_]^–^ solution (pH ∼ 10) show that within 3 h, the Hg^2+^ and Ag^+^ exchange reached equilibrium, with ≥99.9% removal capacities observed (Fig. 10). The fast extraction of Hg^2+^ and Ag^+^ from their cyanide complexes is attributed to the high mobility of the K^+^ ions of KMS-2 and the high affinity of the soft Lewis acids Hg^2+^ and Ag^+^ for the soft basic sulfide framework of KMS-2. It is suggested that KMS-2 absorbs Hg^2+^ and Ag^+^ once the ions are released to the solution and reach equilibrium, according to Le Chatelier's principle (reactions 7–9):

8 [Ag(CN)_2_]^–^ ↔ Ag^+^ + 2CN^–^

9 [Hg(CN)_4_]^2–^ ↔ Hg^2+^ + 4CN^–^

10 K_2_MgSn_2_S_6_ + *x*Hg^2+^ + *y*Ag^+^ → K_2–2*x*–*y*_Hg_*x*_Ag_*y*_MgSn_2_S_6_ + (2*x* + *y*)K^+^

The elemental Hg and Ag can be recovered from the Hg^2+^/Ag^+^-loaded KMS-2 *via* a two step process. First, the exchanged product is heated at 425 °C; HgS sublimes and is collected at the end of a tube which is placed outside the furnace. Then, the Hg-free compound is dissolved in nitric acid, and elemental Ag is isolated by reducing the dissolved Ag^+^ with hydrazine.

Overall, the results from these studies suggest that **MSIEs** are very effective for the recovery of precious metal ions and decontamination of mine wastewater.

#### 2.2.6. UO_2_^2+^ exchange properties

Uranium, which exists primarily as the UO_2_^2+^ cation, represents the major source of nuclear energy and its waste; it can be released into the environment through various industrial and mining processes.^4^ Furthermore, a legacy of uranium-contaminated sites resulted from the closure of various nuclear facilities worlwide.^4^ Selective UO_2_^2+^ ion exchangers to effectively capture this species are thus particularly attractive. In addition to their environmental remediation applications, UO_2_^2+^ sorbents can find use in the extraction of U from seawater, which contains 1000 times more U than terrestrial ores.

Traditionally, suitable sorbents for UO_2_^2+^ are materials with hard basic functional groups,27*a**e.g.* carboxylic, phosphonate, or amidoxime groups, taking into account that U, with a 6+ oxidation state, is expected to behave as a typical hard Lewis acid. However, this may be an oversimplified assumption, since there have been reports of uranyl compounds forming reasonably strong covalent bonds with S^2–^.27*b* KMS-1 shows selective UO_2_^2+^ exchange properties. Complete exchange of the K^+^ ions of KMS-1 by UO_2_^2+^ can be accomplished very fast13*g* according to the following equation:

11 K_1.9_Mn_0.95_Sn_2.05_S_6_ + 0.95UO_2_^2+^ → [UO_2_(H_2_O)_1.5_]_0.95_[Mn_0.95_Sn_2.05_S_6_] + 1.9K^+^

**Fig. 11:**
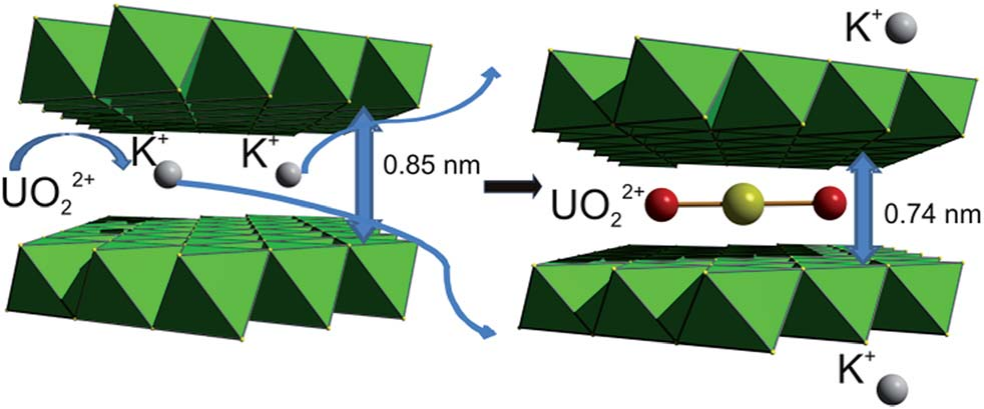
Schematics for the mechanism of UO_2_^2+^ capture by KMS-1.

PXRD studies show that the main UO_2_^2+^-exchanged phase contains the linear [O=U=O]^2+^ group, ordered parallel to the layer plane (Fig. 11).

Solid-state reflectance NIR/UV-Vis data indicated a significantly lower band gap for the UO_2_^2+^ exchanged compound (∼0.95 eV) compared to that (1.3 eV) of pristine KMS-1. This is indicative of relatively strong UO_2_^2+^···S^2–^ interactions.

Isotherm batch sorption data fitted to the Langmuir model reveal a maximum capacity of 380 ± 20 mg g^–1^. The material can be regenerated by treating the UO_2_^2+^-loaded product with Na_2_CO_3_ and can be reused several times for UO_2_^2+^ ion exchange. KMS-1 performs well for UO_2_^2+^ sorption in a broad pH range (2 to 10). Interestingly, very high UO_2_^2+^ removal capacities (94–98%) for KMS-1 were observed even in the presence of tremendous excesses (up to 10^4^ times higher concentration) of competitive ions such as Na^+^ and Ca^2+^.

Furthermore, KMS-1 was tested for UO_2_^2+^ exchange under realistic conditions. It was found to be particularly effective for the decontamination of potable and lake water samples intentionally contaminated with traces of U (400 to 1000 ppb). Thus, the treatment of the samples with KMS-1 for only 2 min results in a final U concentration of ∼1 ppb, well below the acceptable limit (30 ppb, defined by USA-EPA).

KMS-1 has been tested for recovery of U from seawater samples which contain U in concentrations of 3 to 4 ppb and very high amounts of Na^+^, Ca^2+^, Mg^2+^ and K^+^ (200 to 10 000 ppm). KMS-1 efficiently absorbs (removal capacity up to 84%) even this extremely low U content in the presence of reasonably high concentrations of competitive ions. Overall, the results from the UO_2_^2+^ sorption of KMS-1 revealed that this ion is much softer than previously thought, and **MSIEs** may provide new strategies for selective UO_2_^2+^ sorbents. Additional work is required to further evaluate the potential of this material for uranium harvesting from the sea; especially, testing should be performed under more realistic conditions relevant to a scalable practical process.

#### 2.2.7. Cu^2+^ ion-exchange properties

High concentrations of Cu^2+^ in drinking water may result in several health problems, such as liver or kidney damage or gastrointestinal distress.^1^,^28^ KMS-1 was recently tested as a Cu^2+^ exchanger.^29^ The results indicated that Cu^2+^ is inserted as a hydrated cation in the interlayer space, exchanging all K^+^; at the same time, it partially replaces Mn^2+^ ions from the layer. The ion-exchange process is described as follows:

12 K_1.88_Mn_0.94_Sn_2.06_S_6_ + 1.34Cu^2+^ → [Cu(H_2_O)_5.6_]_0.94_[Cu_0.4_Mn_0.54_Sn_2.06_S_6_] + 0.4Mn^2+^ + 1.88K^+^

**Fig. 12:**
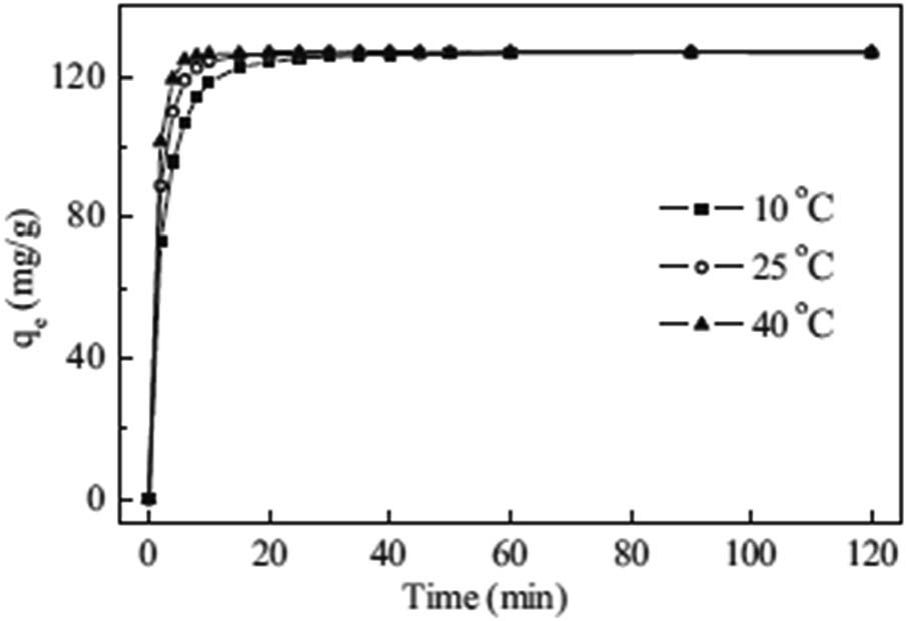
Kinetic data for the sorption of Cu^2+^ by KMS-1, obtained at three different temperatures. Reproduced with permission from ref. 29; © 2014, Elsevier.

A detailed kinetic study has been performed for Cu^2+^ exchange by KMS-1. The data revealed that the absorption of Cu^2+^ is very fast, with ion-exchange equilibrium reached in 40, 20 and 10 min at 10, 25 and 40 °C, respectively (Fig. 12). In addition, fitting of the kinetic data can be readily achieved with the pseudo-second order model, which indicates that the rate limiting step is a chemical adsorption process. Further analysis of the kinetic data revealed that the rate controlling steps were external film and intraparticle diffusion processes.

#### 2.2.8. Sorption of radionuclides

KMS-1 was also tested for the sorption of a series of radionuclides, including ^233^U, ^239^Pu, and ^241^Am.^30^ The results revealed that the material was particularly effective for rapid absorption of these radionuclides over a wide pH range (2 to 9), with ≥99% removal capacities observed even in the presence of high Na^+^ concentrations. This study indicated for the first time that **MSIEs** can be highly efficient for the decontamination of radioactive waste.

### 2.3. Protonated KMS:LHMS material

**Fig. 13:**
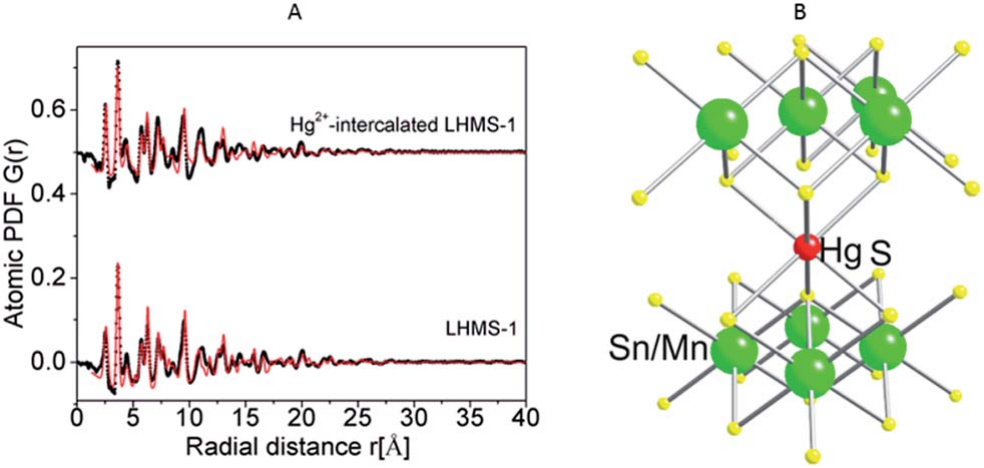
(A) Experimental atomic PDFs (black) for LHMS-1 and Hg^2+^-exchanged material. The red lines represent the computed atomic PDFs based on the SnS_2_ structure type. (B) The octahedral coordination of Hg^2+^ in the exchanged material, which is suggested based on the PDF model.

A rare example of a solid acid metal sulfide material is H_2*x*_Mn_*x*_Sn_3–*x*_S_6_ (*x* = 0.11–0.25), or LHMS-1 (layered hydrogen metal sulfide-1) compound.13*f* This compound is formed from treatment of KMS-1 with highly acidic solution to exchange K^+^ with H^+^, a process which also attests to the high stability of the material to strong acids. LHMS-1 can absorb Hg^2+^ from a very acidic (pH = 0) to strongly alkaline environment (pH = 9). It is remarkable that LHMS-1 achieves almost 100% Hg^2+^ removal under extremely acidic conditions, *e.g.* even in the presence of 6 M HNO_3_. Thus, this compound could be useful for removing Hg^2+^ from acidic wastewater, *e.g.* particular types of nuclear waste.4c,^31^ The local structure of Hg^2+^ in the interlayer space of the Hg^2+^-loaded LHMS material was determined by atomic pair distribution function (PDF) studies, performed for the pristine LHMS-1 material and the Hg^2+^-exchanged product. The results are consistent with an octahedral coordination of Hg^2+^ with Hg–S distances of ∼2.57 Å (Fig. 13).

### 2.4. Layered materials based on the Sn_3_S_7_^2–^ net

**Fig. 14:**
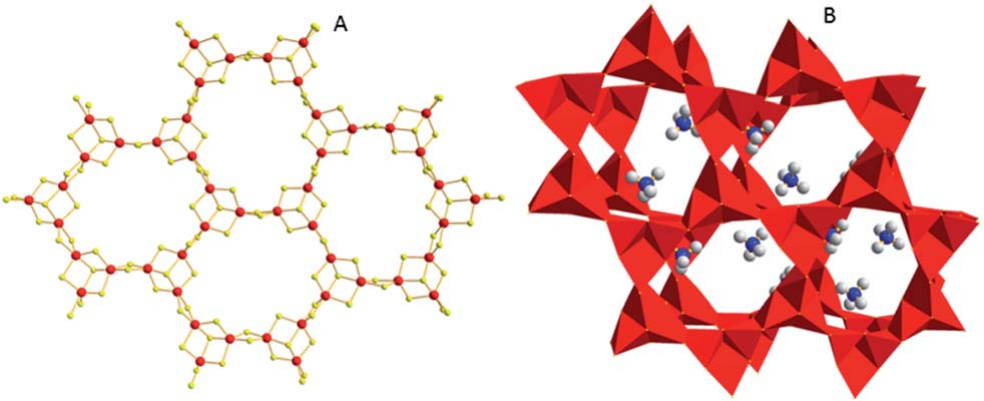
(A) The layered structure of (TMA)_2_(Sn_3_S_7_)·H_2_O (Sn, red; S, yellow). (B) The arrangement of two adjacent layers (with a polyhedral representation) and the TMA^+^ cations (C, grey; N, blue) located in the interlayer space (guest water molecules were omitted for clarity).

Another family of interesting **MSIEs** is based on the Sn_3_S_7_^2–^ layered framework. The first member of this series, (TMA)_2_(Sn_3_S_7_)·H_2_O (TMA^+^ = tetramethylammonium ion), was reported by Prof. J. B. Parise and coworkers.^32^ The compound was isolated by the reaction of SnS_2_, TMAOH and S under hydrothermal conditions. It features a microporous layered framework consisting of edge-sharing [Sn_3_S_4_] semi-cubes (Fig. 14A). Six [Sn_3_S_4_] units are connected *via* S^2–^ bridges to form a ring surrounded by twelve SnS_5_ trigonal bipyramids. The layers are separated by TMA^+^ cations and guest water molecules (Fig. 14B). Preliminary ion-exchange experiments indicated that the TMA^+^ ions are easily exchanged by alkali and alkaline earth ions and some transition metal ions; however, ion exchange with soft metal ions such as Ag^+^ ions resulted in the breakdown of the layered framework structure.

Additional examples of materials with the Sn_3_S_7_^2–^ layered structure contain a variety of interlayer organic cations, such as DABCOH^+^ (protonated 1,8-diazabicyclooctane), QUIN (quinuclidinium), TBA^+^ (*tert*-butylammonium) and Et_4_N^+^ (tetraethylammonium).^33^

**Fig. 15:**
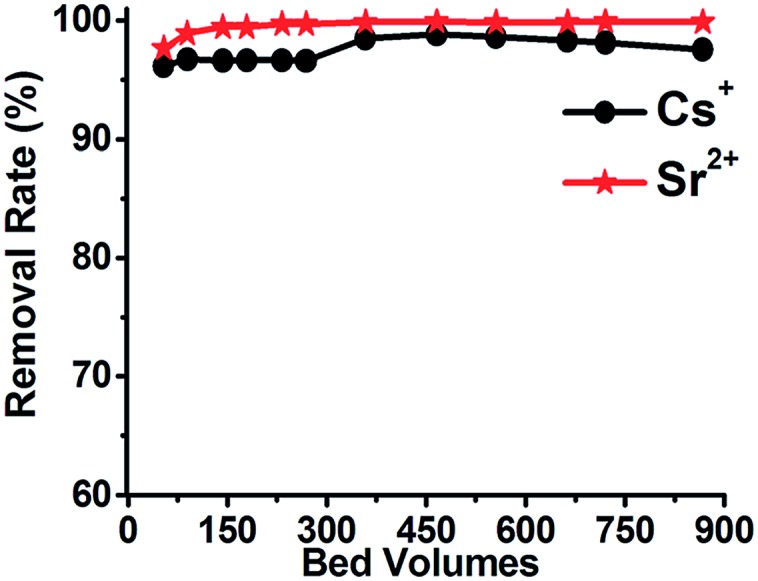
% removal of Sr^2+^ and Cs^+^*vs.* the bed volume for a column of the FJSM-SnS material.

More recently, a new member of the Sn_3_S_7_^2–^ family templated by mixed MeNH_2_^+^ and Me_3_NH^+^ cations was described. The formula of this material, denoted as FJSM-SnS, was (Me_2_NH_2_)_4/3_(Me_3_NH)_2/3_Sn_3_S_7_·1.25H_2_O.^34^ The Cs^+^ and Sr^2+^ exchange properties of this material at 65 °C indicated that the maximum sorption is achieved within only 5 min, whereas at room temperature, the ion exchange equilibrium is reached within 30 to 60 min. The maximum Cs^+^ and Sr^2+^ sorption capacities were 409 ± 29 and 65 ± 5 mg g^–1^, respectively. These values are among the highest reported for **MSIEs**. In addition, pH-dependent experiments revealed good Cs^+^ and Sr^2+^ removal capacities in a broad pH range (0.7 to 10). Interestingly, FJSM-SnS can be used as the stationary phase in an ion-exchange column, which was found to be particularly effective in absorbing Cs^+^ and Sr^2+^ from aqueous solutions containing both ions (initial concentrations: Cs^+^ = 12 to 15 ppm; Sr^2+^ = 6 ppm). Thus, removal capacities of 96–100% Cs^+^ and Sr^2+^ were observed after passing 900 bed volumes (total volume passed = 2.42 L, one bed volume = 2.79 mL) through the ion exchange column (Fig. 15). This represents one of the first reports of the use of **MSIEs** in columns. However, for practical ion-exchange column applications, engineered forms of the sorbents may be required (see below).

### 2.5. KTS materials

**Fig. 16:**
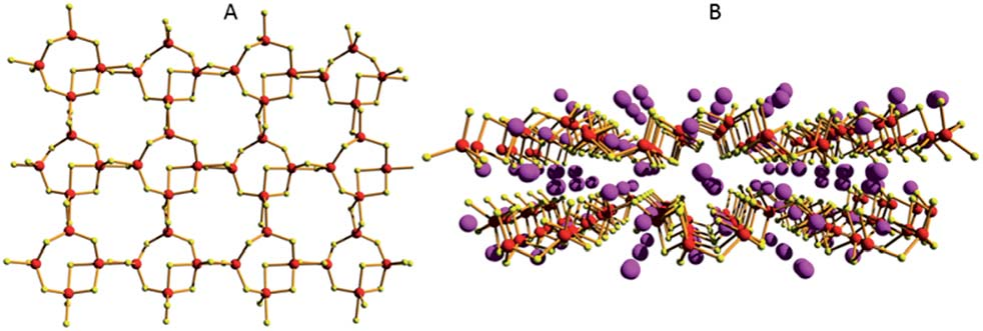
(A) Part of the Sn_4_S_9_^2–^ layer (Sn, red; S, yellow). (B) The arrangement of two adjacent layers with the cations (Cs^+^, pink) filling the interlayer space.

An additional example of a tin sulfide layered material with promising ion-exchange properties is K_2_Sn_4_S_9_ (KTS-1). This compound can be isolated *via* solid state synthesis. The crystal structure of KTS-1 is related to the structures of Rb_2_Sn_4_S_9_ and Cs_2_Sn_4_S_9_.^35^ The basic unit of the structure of these materials is the Sn_4_S_9_^2–^ duster, consisting of two tetrahedrally coordinated Sn^4+^ ions and two Sn^4+^ ions adopting trigonal bipyramidal geometry. The clusters are connected through S^2–^ bridges to form a layered structure perforated with relatively large holes (Fig. 16A). The layers are corrugated, and the interlayer space is filled with highly disordered cations (Fig. 16B).

Preliminary investigations of the Cs^+^ exchange properties of KTS-1 revealed a maximum sorption capacity of 205 ± 6 mg g^–1^ and very high *K*_d_ values in the range of 10^3^ to 10^5^ mL g^–1^. Furthermore, the K^+^ ions of KTS-1 can be fully exchanged by soft metal ions, such as Hg^2+^, Pb^2+^ and Cd^2+^.^36^ Thus, KTS-1 appears to be a promising ion-exchanger, although further studies are required.

KTS-2, another tin sulfide ion-exchanger, is a 3-D material and is discussed below (see Section 2.3).^36^

**Fig. 17:**
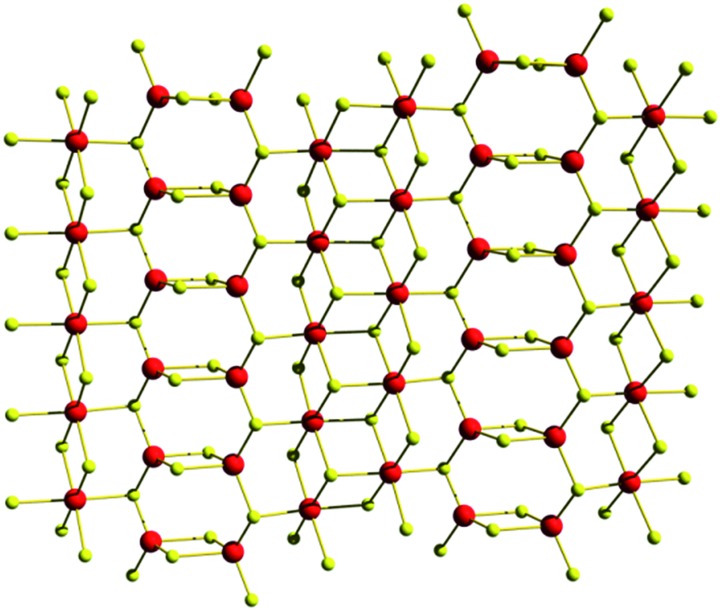
Structural segment of the KTS-1 layer viewed along the *b*-axis. Sn, red; S, yellow.

Very recently, a new compound, K_2*x*_Sn_4–*x*_S_8–*x*_ (*x* = 0.65 to 1, KTS-3) was reported.13*i* This compound was prepared with a hydrothermal reaction similar to that used for KMS materials, but without adding Mn or Mg to the reaction mixture. The crystal structure of KTS-3 is shown in Fig. 17. SnS_6_ octahedra form ribbons running along the c-axis and are interconnected through SnS_4_ units in the form of Sn_2_S_6_ bridges. The interlayer space is filled by disordered K^+^ ions.

**Fig. 18:**
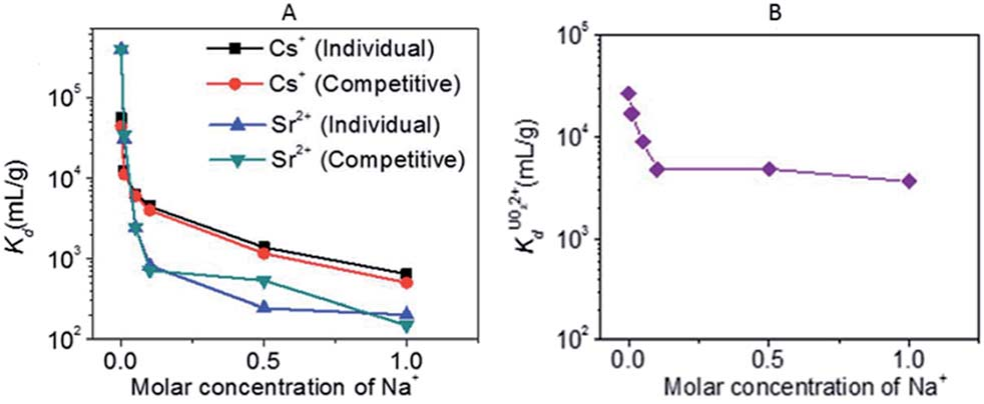
(A) *K*_d_ of individual and competitive Cs^+^ and Sr^2+^ ion exchange *vs.* molar concentration of Na^+^ (initial concentrations were 7.4 and 6.9 ppm for Cs^+^ and Sr^2+^ respectively, V m^–1^ ratio was 1000 mL g^–1^, and pH ∼ 7). (B) *K*_d_ for UO_2_^2+^ ion exchange *vs.* molar concentration of Na^+^ (initial concentration of UO_2_^2+^ was 1 ppm, V m^–1^ ratio was 1000 mL g^–1^, and pH ∼ 7).

KTS-3 is an excellent ion-exchanger for Cs^+^, Sr^2+^ and UO_2_^2+^. The Sr^2+^ capacity of this sorbent was found to be 102 ± 5 mg g^–1^ (by fitting of the data with the Langmuir model), the highest reported among **MSIEs**. It also shows high Cs^+^ and UO_2_^2+^ exchange capacities (Langmuir fitting: 280 ± 11 mg Cs/g, 287 ± 15 mg U/g). Interestingly, it exhibits very good selectivity for Cs^+^ (*K*_d_ value of 4.4 × 10^3^ mL g^–1^) in the presence of Na^+^ in a molar concentration (0.1 M) 2000 times higher than the initial Cs^+^ content (Fig. 18A). Even with a 10 000-fold excess of Na^+^, the *K*_d_ value for Cs^+^ was >10^3^ mL g^–1^. The effect of Na^+^ seems to be more important in the case of ion-exchange of Sr^2+^; the *K*_d_ values dropped sharply when the Na^+^ concentration was increased (Fig. 18B). Furthermore, competitive Cs^+^/Sr^2+^ ion exchange experiments revealed generally higher *K*_d_ values for Cs^+^ exchange than Sr^2+^ exchange (Fig. 18A). Finally, Na^+^ even in huge excess (concentration up to 5 M) has a negligible effect on UO_2_^2+^ exchange because the *K*_d_ values show only slight variation when the Na^+^ concentration is increased (Fig. 18B). This result further indicates that UO_2_^2+^ behaves more like a typical soft ion (borderline soft) interacting strongly with the soft basic metal sulfide layer, rather than a hard ion, as UO_2_^2+^ is generally classified. KTS-3 is also an effective sorbent for heavy metals such as Hg^2+^, Pb^2+^, Cd^2+^, Ag^+^, and Tl^+^.

### 2.6. Layered sulfides with trivalent metal ions in their framework

The majority of layered **MSIEs** are based on tetravalent or combined tetravalent/bivalent metal ions. Generally, these metal ions mainly adopt tetrahedral or octahedral coordination; in only a few cases, trigonal bipyramidal coordination has been also observed. An alternative approach to create sulfide materials involves the combination of trivalent ions such as In^3+^ or Ga^3+^, which prefer tetrahedral coordination, and Sb^3+^, which tends to adopt a trigonal pyramidal coordination geometry.

#### 2.6.1. [(CH_3_CH_2_CH_2_)_2_NH_2_]_5_In_5_Sb_6_S_19_·1.45H_2_O (InSbS)

**Fig. 19:**
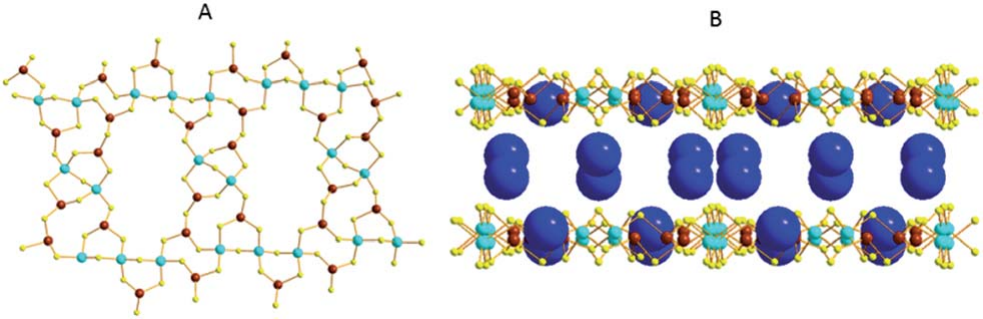
(A) Part of the [In_5_Sb_6_S_19_]^5–^ layer viewed down the *b*-axis. (B) Packing of the layers with the N atoms of dipropylammonium cations showing as large balls (C and H atoms were omitted for clarity). In, cyan; Sb, brown; N, blue; S, yellow.

A layered material isolated with this synthetic strategy is [(CH_3_CH_2_CH_2_)_2_NH_2_]_5_In_5_Sb_6_S_19_·1.45H_2_O (InSbS), prepared by a hydrothermal reaction of In_2_S_3_, Sb_2_S_3_, S and dipropylamine.^37^ The [In_5_Sb_6_S_19_]^5–^ layer is composed of corner-sharing InS_4_ tetrahedra bridged by SbS_3_ trigonal pyramidal units and Sb_2_S_6_ dimers (Fig. 19A). The layer contains large holes with dimensions 13.3 × 3.8 Å^2^, which are large enough to host some of the organic counter ions, with the remaining organic cations located in the interlayer space (Fig. 19B).

Due to the disordered state of the organic guest ions and the relatively large windows of the layer, this compound displays facile ion-exchange properties with various cationic species. Interestingly, Cs^+^ can completely exchange the organic cations, whereas Rb^+^ exchanges only 37% of the cations. In addition, 12% of ions are replaced by Li^+^, and a negligible amount of organic guests can be exchanged by K^+^ and Na^+^.

Competitive experiments with a mixture of equimolar amounts of Na^+^, K^+^, Rb^+^ and Cs^+^ revealed that the Cs^+^ uptake of InSbS is 10 times higher than that for the other ions. This significant selectivity of the compound for Cs^+^ probably results from the size-match of this cation with the aperture of the windows in the [In_5_Sb_6_S_19_]^5–^ layer. Thus, the perforated layers of the compound seem to favor its facile ion-exchange properties. This hypothesis is also supported by the lack of any ion-exchange capacity for the lamellar material [CH_2_NH_2_]_2_In_2_Sb_2_S_7_, whose layers are relatively dense and have no holes.

#### 2.6.2. [CH_2_NH_2_]_2_Ga_2_Sb_2_S_7_·H_2_O (GaSbS-1)

**Fig. 20:**
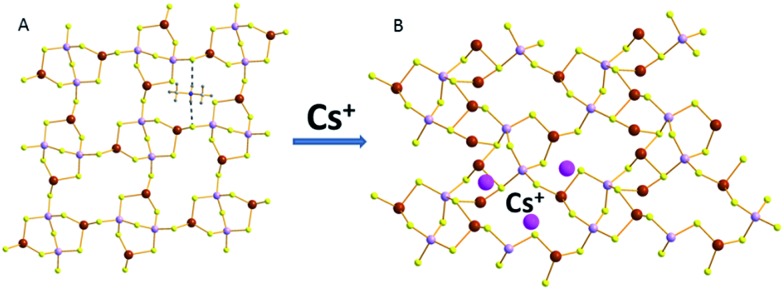
(A) Part of the layer of GaSbS-1. The dashed lines represent hydrogen bonding interactions between the dimethylammonium ions and the layer. (B) Part of the layer of GaSbS-2 and some of the interlayer Cs^+^ ions. Ga, purple; Sb, brown; S, yellow; Cs, pink.

Another example of a layered MSIE material containing trivalent metal ions is [CH_2_NH_2_]_2_Ga_2_Sb_2_S_7_·H_2_O (GaSbS-1).^38^ The building block of the structure is made of two corner-sharing GaS_4_ tetrahedra and two SbS_3_ trigonal pyramidal units further bridging the GaS_4_ moieties (Fig. 20A). There are relatively open windows in the layers that are defined by 16-member rings composed of four building units. Dimethylammonium cations are found in the interlayer space and interact with the layers *via* N–H···S hydrogen bonding.

GaSbS-1 shows facile ion-exchange properties for alkali and alkaline earth metal ions due to its layered structure and the relatively large windows in the layers, which allow the diffusion of species in the ion-exchange reactions. Of particular interest is Cs^+^ sorption by the compound GaSbS-1. This material is particularly selective for Cs^+^ in the presence of a very large (100-fold) excess of Na^+^. Interestingly, the Cs^+^ exchange can be achieved even *via* a SCSC transformation process. The crystal structure of the Cs^+^-exchanged product (GaSbS-2) could be thus determined.

**Fig. 21:**
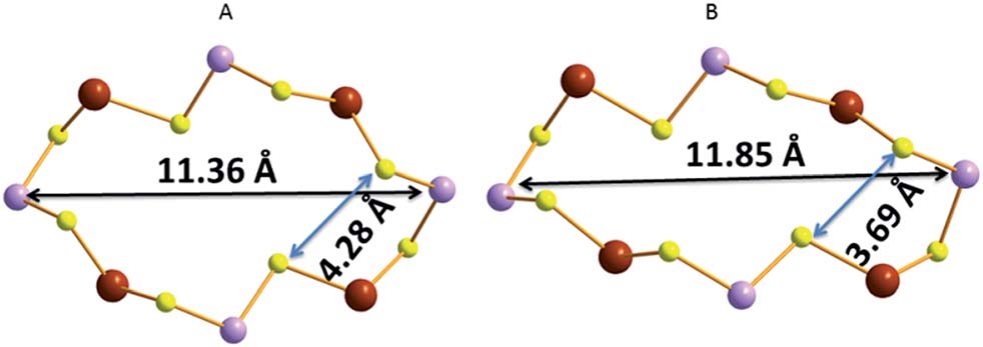
(A) The 16-member ring in GaSbS-1 with indication of its dimension. (B) The 16-member ring in GaSbS-2 with indication of its dimension. Ga, purple; Sb, brown; S, yellow..

The connectivity of the atoms in GaSbS-2 (Fig. 20B) is the same as in GaSbS-1. There is, however, a significant contraction of the layered framework. Thus, the size of the windows changed from 11.36 × 4.28 Å^2^ in GaSbS-1 to 11.85 × 3.69 Å^2^ (including atomic radii) in GaSbS-2 (Fig. 21). Therefore, it appears that there is a framework response when Cs^+^ ions enter the structure, resulting in shrinkage of the window size in the layers. Two of the three crystallographically independent Cs^+^ ions are located close to the layer, whereas the third one is found between two layers. These ions are tightly bound in the compound, as no Cs^+^ can be leached out by treating the material with large excess of Na^+^ ions. The opening/closing of windows observed for the layered structure of compound GaSbS-1 upon Cs^+^ exchange is reminiscent of the mechanism of insect capture by the Venus flytrap plant.

#### 2.6.3. [CH_3_NH_3_]_20_Ge_10_Sb_28_S_72_·7H_2_O (GeSbS-1)

**Fig. 22:**
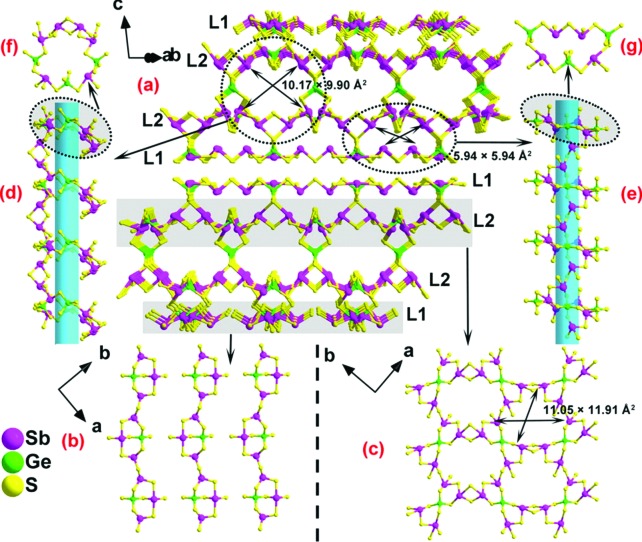
(a) The double-layered network of GeSbS-1. (b) The L1 unit. (c) The L2 unit. (d and e) The two types of channels in the network. (f and g) The 18- and 14-membered rings that form the channels in the structure. Reproduced with permission from ref. 39 © 2015, American Chemical Society.

This compound was prepared solvothermally by reacting GeO_2_, Sb, S and methylammonium salt.^39^ It features an unusual double-layered structure formed by two symmetry-related thick layers joined by Ge^4+^ ions (Fig. 22). Each single layer is composed of two parts, L1 and L2. L1 is a 1-D chain of interconnecting [GeSb_3_S_8_]^3–^ units, whereas the L2 moiety is a layer based on [GeSb_4_S_11_]^6–^ units. Two types of channels are observed in the structure, with dimensions of 10.17 × 9.90 Å^2^ and 5.94 × 5.94 Å^2^. The methylammonium cations and guest water molecules are found within these channels as well as in the interlayer space.

GeSbS-1 shows impressive Cs^+^ exchange properties. Specifically, it exhibits a Cs^+^ exchange capacity of 231 ± 15 mg g^–1^. The kinetics of the Cs^+^ sorption is also remarkably fast, with the ion exchange reaching equilibrium within 2 min. In addition, the selectivity of the material for Cs^+^ is significant, as revealed by the relatively high *K*_d_ values (2 to 4 × 10^3^ mL g^–1^) for Cs^+^ exchange in the presence of a large excess (20-fold) of Na^+^ or Ca^2+^.

### 2.7. Polysulfide and MoS_4_^2–^-intercalated layered double hydroxides

**Fig. 23:**
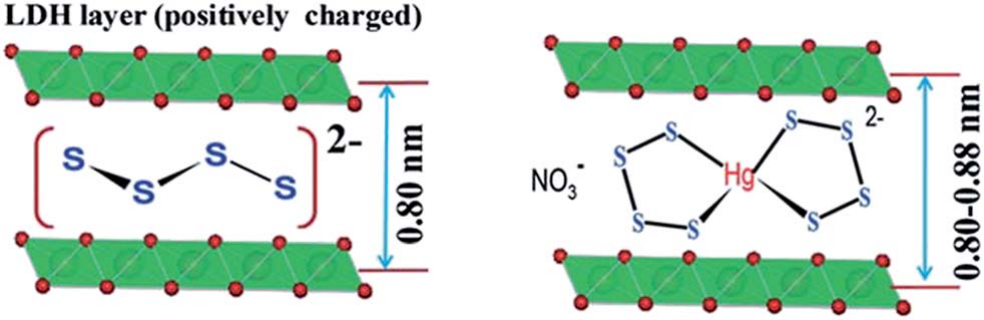
Left: Arrangement of [S_4_]^2–^ groups in the interlayer space of the LDH material. Right: Proposed binding of metal ions with the polysulfide unit. Metal, green; O, red.

Recently, a new type of metal sulfide-like sorbent, with selective sorption properties for heavy metal ions, has been developed.^40^ These materials consist of layered double hydroxides (LDHs) intercalated by polysulfide [S_*x*_]^2–^ groups (Fig. 23, left).

The mechanism of the metal ion capture by LDH(S_*x*_) sorbents depends on the metal ion : LDH(S_*x*_) molar ratio.

In the case of low metal ion concentration and a large excess of sorbent, the following reaction takes place:

13 LDH(S_*x*_) + M(NO_3_)_2_ → LDH(NO_3_^–^)_*m*_([M^2+^(S_*x*_^2–^)_2_]^2–^)_*n*_

Thus, the [S_*x*_]^2–^ groups act as a second host for the incoming ions (Fig. 23, right).

When, however, the concentration of the metal ion is relatively large, the sorption reactions are stoichiometric; thus, two products, LDH(NO_3_) and MS_*x*_, are formed:

14 LDH(S_*x*_) + M(NO_3_)_2_ → LDH(NO_3_) + MS_*x*_

Sorption experiments with aqueous solutions containing a series of metal ions, such as Ni^2+^, Co^2+^, Cu^2+^, Cd^2+^, Hg^2+^, Pb^2+^, Zn^2+^ and Ag^+^, indicate higher selectivity of LDH(S_*x*_) for the softer ions. Extremely high *K*_d_ values (up to 10^7^ mL g^–1^) are found for the sorption of Ag^+^ and Hg^2+^, revealing the high potential of LDH(S_*x*_) sorbents for the capture of heavy metal ions.40*a*

LDH(S_*x*_) materials were also tested for UO_2_^2+^ sorption.40*b* A series of UO_2_^2+^ sorption experiments reveal the exceptional selectivity of the LDH(S_*x*_) sorbents for UO_2_^2+^ in the presence of very large excesses of Na^+^ and Ca^2+^. In addition, LDH(S_*x*_) materials can efficiently absorb U from seawater samples. These results further confirm the potential of sulfide sorbents for selective U capture.

More recently, MoS_4_^2–^ intercalated LDHs have been reported.40*c* They show extremely high sorption capacities for Hg^2+^ (∼500 mg g^–1^) and Ag^+^ (∼450 mg g^–1^). They are also efficient to capture additional metal ions such as Cu^2+^, Pb^2+^, and Co^2+^. It is suggested that in low and medium metal (M^2+^) ion concentrations, the sorption proceeds through the formation of [M(MoS_4_)_2_]^2–^ species trapped in the interlayer space of the LDH materials. In high initial metal ion concentrations, neutral amorphous [M(MoS_4_)] salts are produced.

## 3. Three-dimensional crystalline MSIEs

Although a large number of three-dimensional crystalline metal sulfide materials have been reported, relatively few examples have been thoroughly studied for their ion-exchange properties. Below, we describe some characteristic 3-D **MSIEs** and their sorption properties for various cations.

### 3.1. K_6_Sn[Sn_4_Zn_4_S_17_] (K_6_MS)

**Fig. 24:**
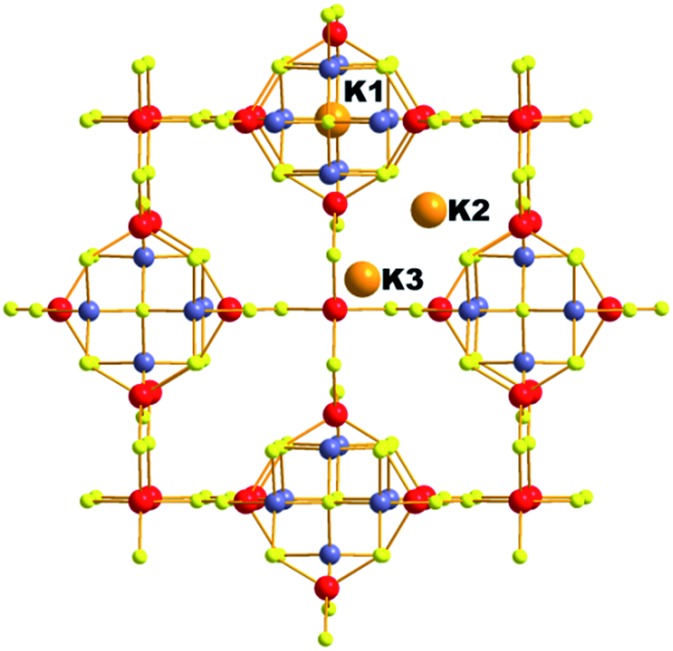
The three-dimensional framework of K_6_MS with labeling of the K^+^ ions. Sn, red; Zn, blue-grey; S, yellow.

**Fig. 25:**
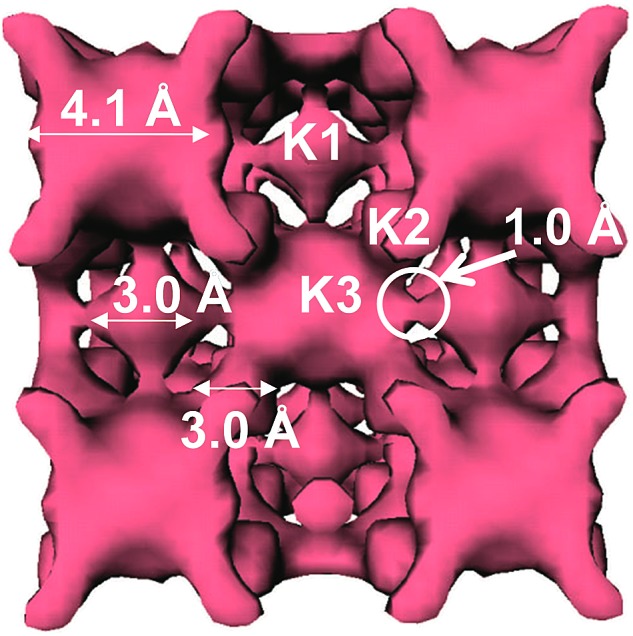
Plot of the void space of K_6_MS with labeling of the three different types of pores and indication of the sizes of the cavities.

This compound was isolated *via* solid state flux synthesis.^41^ Its structure is based on the so-called penta-supertetrahedral (P1) [Zn_4_Sn_4_S_17_]^10–^ cluster, constructed by a central {Zn_4_S}^6+^ core (anti-T1 unit) capped by four SnS_4_ tetrahedral (T1) units. The clusters are interconnected *via* four-coordinated Sn^4+^ ions to form a diamond-like framework (Fig. 24).13*a* There are 3 cavities in the structure which host K^+^ ions. The K_1_ cavity accommodates a tightly bound K^+^ ion (K_1_), whereas K_2_ and K_3_ cavities contain four (K_2_) and one (K_3_) K^+^ ions, respectively, which are relatively labile. The K_3_ cavity has a relatively large diameter (∼4.1 Å) and communicates through narrow passages (with sizes of ∼1.0 to 1.5 Å) with the smaller K_1_ and K_2_ pores (with a diameter of ∼3.0 Å) (Fig. 25).

#### 3.1.1. Cs^+^ ion-exchange properties

**Fig. 26:**
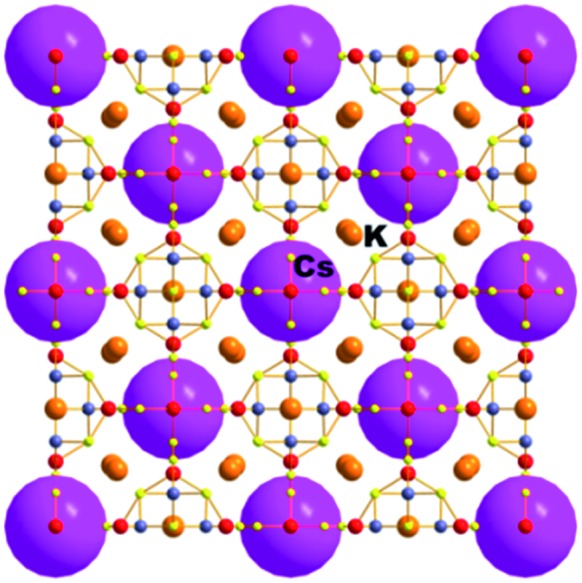
Representation of the structure of K_5_CsSn[Zn_4_Sn_4_S_17_]. Sn, red; Zn, blue-gray; S, yellow.

K_6_MS shows interesting Cs^+^ ion-exchange properties, which may be observed with either polycrystalline samples or single crystals of the material (**SCSC** transformation).13*b* Reaction of K_6_MS with CsCl for ∼12 h gives K_5_CsSn_5_Zn_4_S_17_. The **SCSC** Cs^+^ ion-exchange reaction resulted in the isolation of single crystals of the Cs^+^-exchanged product. Determination of its crystal structure revealed the presence of one Cs^+^ ion in the place of K_3_^+^ of K_6_MS (Fig. 26). For comparison, the corresponding **SCSC** Rb^+^ ion exchange led to the isolation of a product with 5 Rb^+^ replacing K_2_^+^ and K_3_^+^ ions (only K_1_^+^ remained intact). Competitive Rb^+^/Cs^+^ ion-exchange reactions using a large excess of Rb^+^ (Rb : Cs molar ratio = 10 : 1) yielded a material with three K^+^, two Rb^+^ and one Cs^+^ according to analytical data (the crystal structure was not determined). Despite the high concentration of Rb^+^ related to that of Cs^+^, the material still absorbs one Cs^+^ per formula unit, thus indicating its strong selectivity for this ion.

**Fig. 27:**
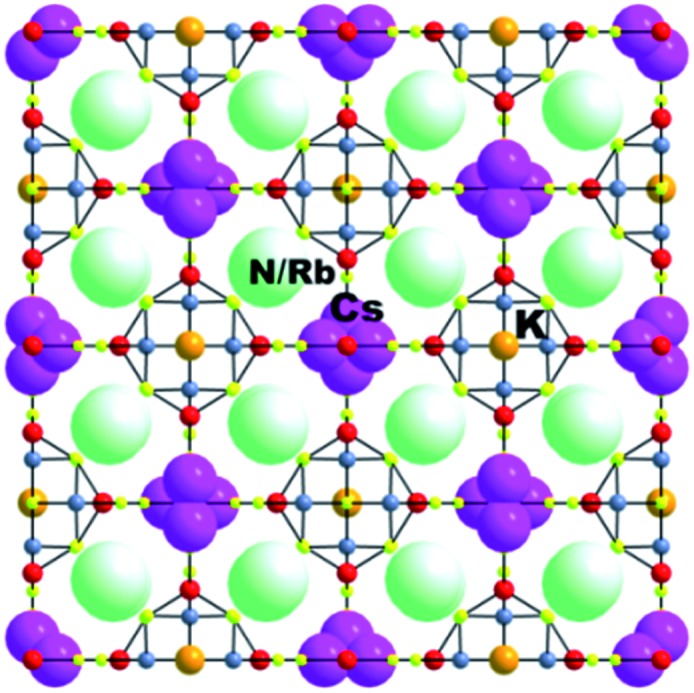
Representation of the structure of KCsRb_1.29_(NH_4_)_2.71_Sn[Zn_4_Sn_4_S_17_]. Sn, red; Zn, blue-gray; S, yellow.

**Scheme 1:**
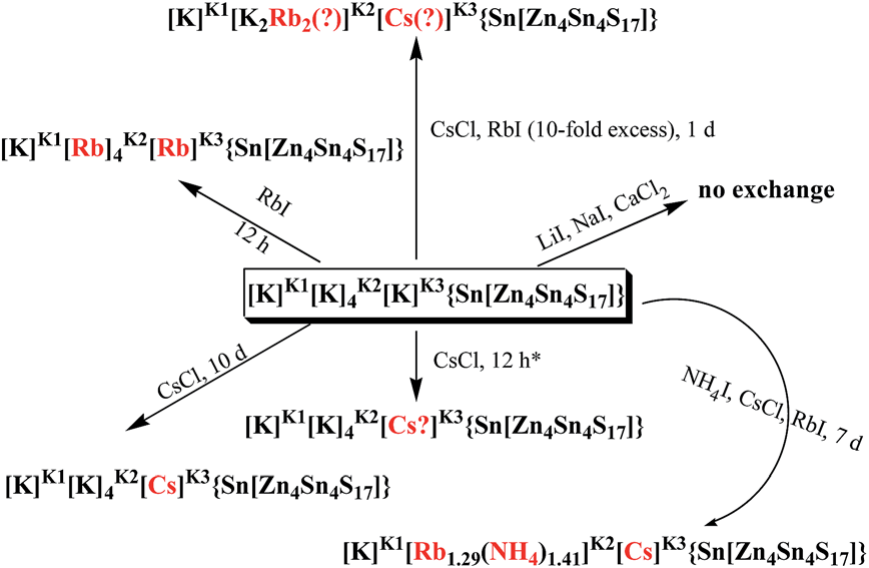
Representation of the ion-exchange reactions for K_6_MS.

An **SCSC** exchange experiment was also performed in the simultaneous presence of Cs^+^, Rb^+^ and NH_4_^+^ in equimolar concentrations. The results revealed that the exchange product contained only K^+^ in its K_1_ cavity, the K_2_ cavity hosted a mixture of Rb^+^/NH_4_^+^ and the K_3_ cavity was filled exclusively with Cs^+^ (Fig. 27). Therefore, the K_3_ cavity seems to be suitably sized for Cs^+^, which explains the selectivity of K_6_MS for this ion. Interestingly, K_6_MS shows absolutely no exchange capacity for Li^+^, Na^+^ and Ca^2+^ because the large hydration sphere of these ions prevents them from entering the framework. The ion-exchange reactions for K_6_MS are summarized in Scheme 1.

**Fig. 28:**
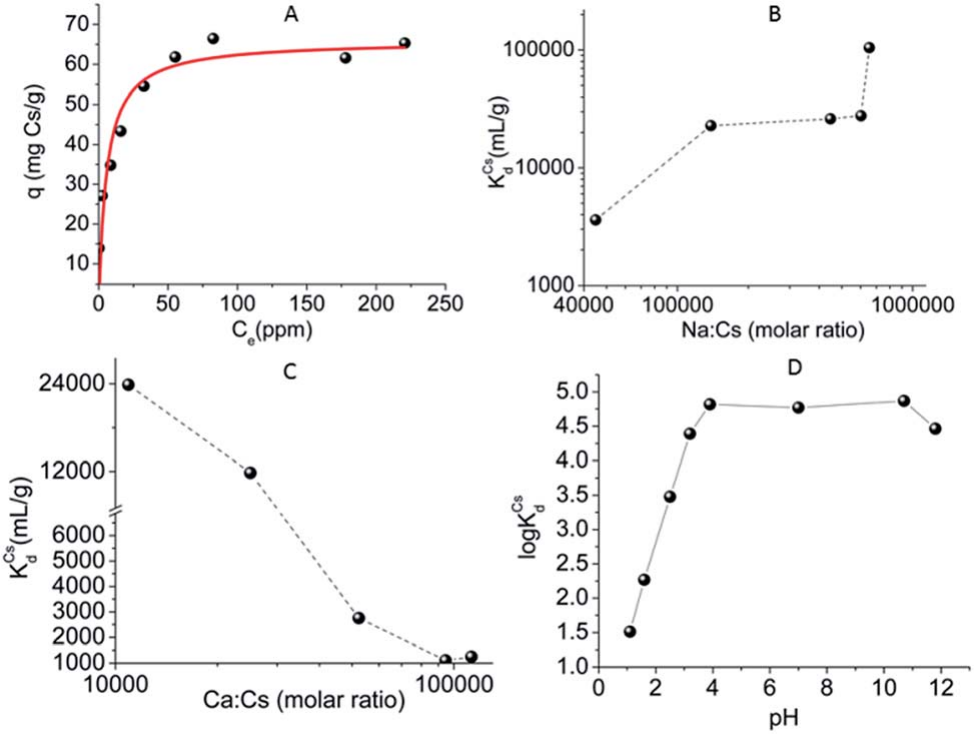
(A) Isotherm Cs^+^ sorption data for K_6_MS. The solid line represents the fitting of the data with the Langmuir model (*q*_m_ = 66 ± 4 mg g^–1^; *b* = 0.17 ± 0.05 L mg^–1^, *R*^2^ = 0.89). (B) The distribution coefficient *K*_d_ for Cs^+^ ion exchange *versus* the Na^+^ : Cs^+^ molar ratio. (C) The distribution coefficient *K*_d_ for Cs^+^ ion exchange *versus* the Na^+^ : Cs^+^ molar ratio. (D) The logarithm values of the distribution coefficient *K*_d_ for Cs^+^ ion exchange *versus* the pH.

Encouraged by the above excellent Cs^+^ exchange results, more detailed batch ion-exchange studies have been performed.^42^ Ion-exchange isotherm data, which fit the Langmuir model (Fig. 28A), reveal a maximum Cs^+^ ion-exchange capacity of 66 ± 4 mg g^–1^, corresponding to 0.81 ± 0.06 mole of Cs^+^ per formula unit, *i.e.* close to the expected maximum Cs^+^ capacity (1 mole per formula unit).

The sorption of low concentration (∼1 ppm) Cs^+^ in the presence of extremely high concentrations of Na^+^ (0.4 to 5 M) is exceptional. Specifically, >95% Cs^+^ sorption and large distribution coefficients *K*_d_ in the range of 10^4^ to 10^5^ mL g^–1^ were estimated, even with 1.3 to 6.5 × 10^5^-fold excesses of Na^+^ (Fig. 28B). Surprisingly, an enhancement of the Cs^+^ sorption by K_6_MS was observed upon increasing the Na^+^ concentration.

Very high Ca^2+^ concentrations (0.1 to 1 M) partially reduce the Cs^+^ sorption capacity of K_6_MS. High Cs^+^ removal capacity (∼68%) and distribution coefficients above 1000 mL g^–1^ were observed even in the presence of a 10^5^-fold excess of Ca^2+^ (Fig. 28C).

K_6_MS also exhibits excellent affinity and selectivity for Cs^+^, showing *K*_d_ values ≥10^4^ mL g^–1^ within a very wide pH range (3 to 12), Fig. 28D. These data indicate that K_6_MS may be effective for Cs^+^ decontamination for both alkaline and acidic waste solutions. The above results confirm the exceptional selectivity of K_6_MS for Cs^+^, which results from the perfect fit of the K_3_ cavity for Cs^+^ (see above).

#### 3.1.2. NH_4_^+^ ion-exchange properties

This material also exhibited very interesting NH_4_^+^ exchange properties.13*b* Treating single crystals of compound K_6_MS with NH_4_I for 1 week resulted in the isolation of an exchange product with 5 NH_4_^+^ and only one K^+^ per formula unit. Surprisingly, NH_4_^+^ replaced all K^+^ except the highly disordered K_3_^+^ ion. It is remarkable that NH_4_^+^ can diffuse even through the very narrow passages connecting the K_1_ and K_3_ cavities. This highly unusual NH_4_^+^ exchange capability of K_6_MS is attributed to the flexibility of the framework of this material. The M–S–M′ angles in K_6_MS are relatively small (∼110°), which facilitates breathing phenomena and, thus, the movement of ions through the tunnel network. This behavior of K_6_MS is in marked contrast to that of zeolites, which are characterized by wide Al–O–Si angles (160 to 180°) and do not favor swelling processes to the same degree.

#### 3.1.3. Heavy metal ion sorption properties

**Fig. 29:**
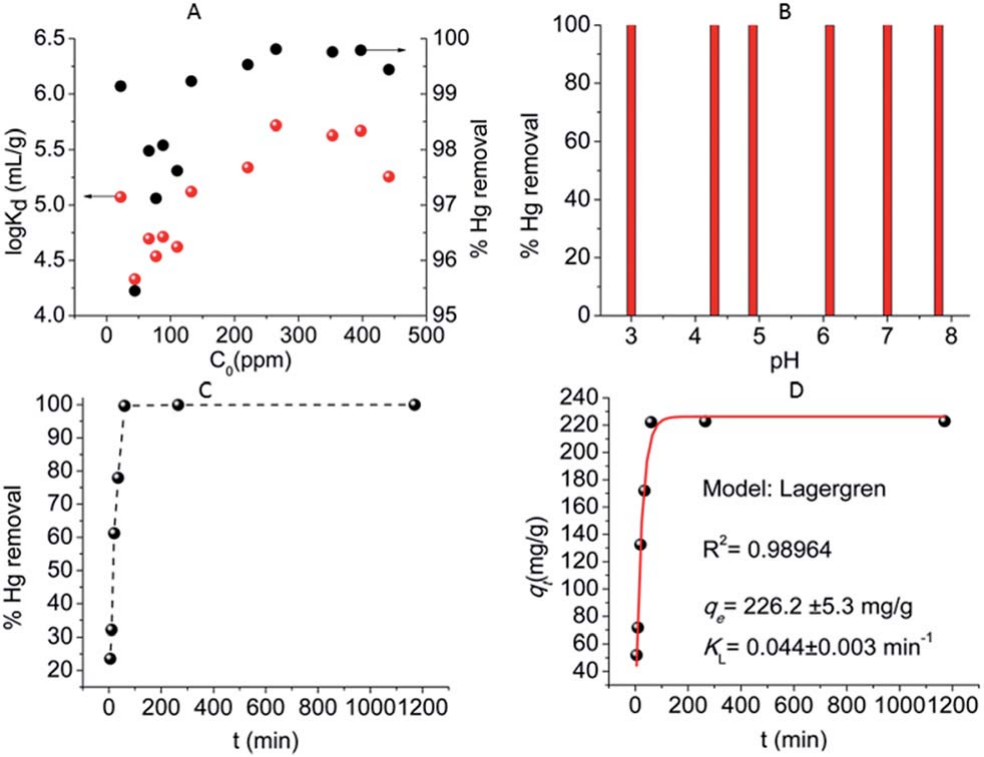
(A) Representation of the % Hg removal and log *K*_d_ values *versus* the initial Hg^2+^ concentration for K_6_MS. (B) % Hg removal *vs.* pH (initial Hg^2+^ concentration = 441 ppm, V m^–1^ = 500 mL g^–1^). (C) % Hg removal *vs.* time (min) (initial Hg^2+^ concentration = 441 ppm, V m^–1^ = 500 mL g^–1^). (D) Fitting of the kinetic data with Lagergren's equation (red solid line).

K_6_MS selectively absorbs soft metal ions.^43^ Hg^2+^ exchange experiments with various initial Hg^2+^ concentrations indicated very high removal capacities (95–100%) and *K*_d_ values of up to 2 × 10^6^ mL g^–1^ (Fig. 29A). In addition, experiments at various pH values (3 to 8) revealed no effect of the pH on the Hg^2+^ sorption, which reaches almost 100% removal capacity over the whole pH range tested (Fig. 29B). The investigation of kinetics for the Hg^2+^ ion exchange (initial Hg^2+^ concentration ∼441 ppm, pH ∼ 5) revealed that equilibrium is reached within only one hour (Fig. 29C). Fitting of the kinetic data was performed with the Lagergren's equation describing first-order kinetics:

15 *q*_*t*_ = *q*_e_[1 – exp(–*K*_L_*t*)],

where *q*_e_ = the amount (mg g^–1^) of metal ion absorbed in equilibrium, *K*_L_ = the Lagergren or first-order rate constant (min^–1^).12*b* The fitting (Fig. 29D) indicated a maximum Hg^2+^ sorption capacity of 226 ± 5 mg g^–1^ (for an initial Hg^2+^ concentration of 441 ppm) and a rate constant of 0.044 ± 0.003 min^–1^.

Extremely high Na^+^ and Ca^2+^ concentrations (1 to 5 M) had no influence on the Hg^2+^ sorption by K_6_MS, and the *K*_d_ values obtained under these conditions were ≥10^6^ mL g^–1^.

K_6_MS can efficiently remove a variety of other heavy metal ions, such as Pb^2+^, Cd^2+^, Ag^+^ and Tl^+^. Specifically, K_6_MS absorbed 98.7–99.7% each of Hg^2+^ (166 ppm), Pb^2+^ (67 ppm) and Cd^2+^ (40 ppm) from a solution containing all three ions. Furthermore, K_6_MS exhibited very high *K*_d_ values (up to 10^7^ mL g^–1^) for Ag^+^ exchange.

**Fig. 30:**
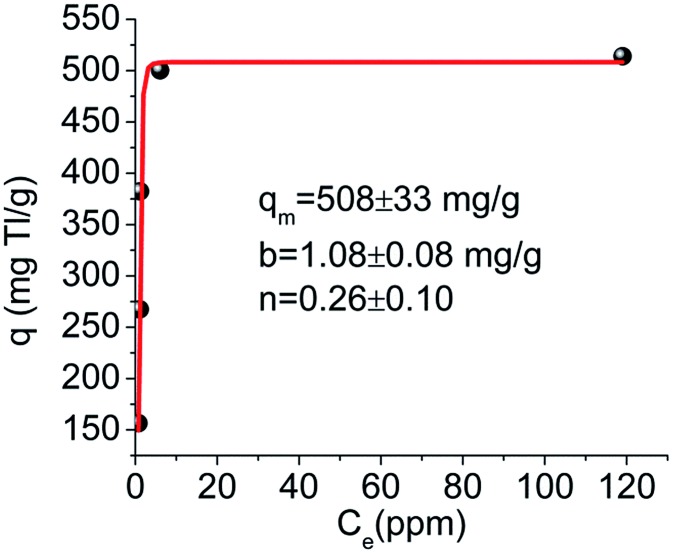
Isotherm Tl^+^ sorption data for K_6_MS (pH ∼ 7). The solid line represents the fitting of the data with the Langmuir Freundlich model.

K_6_MS was also highly efficient for the removal of Tl^+^. This ion presents very high toxicity, and its removal from contaminated water resources is of high priority.^44^ Isotherm Tl^+^ exchange data (Fig. 30) for K_6_MS can be fitted with the Langmuir–Freundlich model:
16

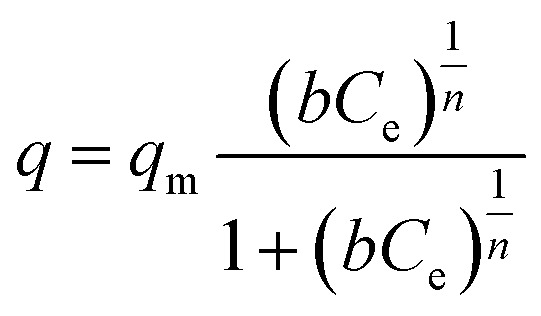

where *n* and *b* (L mg^–1^) are constants and *q*_m_ (mg g^–1^) is the maximum sorption capacity at the equilibrium concentration *C*_e_ (ppm).^13^

The fitting indicated a maximum sorption capacity of 508 ± 33 mg Tl g^–1^, which is consistent with the absorption of ∼4 moles of Tl^+^ per formula unit of K_6_MS. *K*_d_ values for Tl^+^ exchange were found high (up to 2.2 × 10^5^ mL g^–1^) revealing the high affinity of K_6_MS for Tl^+^.

### 3.2. [(Me)_2_NH_2_]_2_[Sb_2_GeS_6_] (GeSbS-2)

**Fig. 31:**
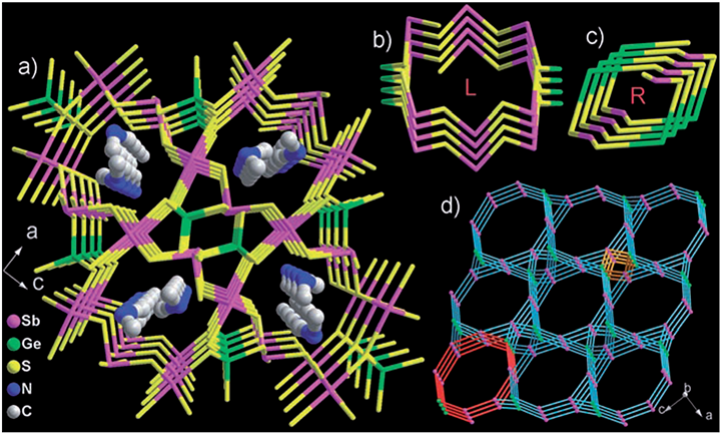
(a) Structure of [(Me)_2_NH_2_]_2_[Sb_2_GeS_6_] along the *b*-axis. (b) The left-handed helical chain parallel to the *b*-axis. (c) The right-handed helical chain parallel to the *b*-axis. (d) The (3^2^ × 10^4^) net topology of the structure. The left-handed and right-handed helical chains are highlighted with red and orange, respectively. Reproduced with permission from ref. 45; © 2008, Wiley-VCH.

This compound was isolated *via* a solvothermal reaction of GeO_2_, Sb and S in DMF.^45^ The structure is chiral and based on the interconnection of helical chains (Fig. 31A). Specifically, there are two types of chains: one chain is left-handed, formed by [Sb_3_S_10_] units and GeS_4_ tetrahedra (Fig. 31B). The second chain is right-handed constructed by GeS_4_ units linked with the middle trigonal bipyramidal SbS_4_ moieties (Fig. 31C). The Me_2_NH_2_^+^ ions are located in the pores forming N–H···S hydrogen bonds with the framework (Fig. 31A). From the topological point of view, the structure can be described as a 4-connected net [(3^2^ × 10^4^) net topology], with both GeS_4_ and SbS_4_ units acting as 4-connected nodes (Fig. 31D).

The compound shows ion-exchange properties for alkali metal ions. The Cs^+^ ion-exchange study indicated that ∼93% of the organic cations can be replaced with Cs^+^. The crystal structure of the Cs^+^-exchanged compound was also determined indicating topotactic ion-exchange. The material shows high selectivity for Cs^+^, as revealed by competitive Cs^+^–Na^+^ exchange studies. Thus, the ion-exchange reaction with a Na^+^ : Cs^+^ molar ratio of 10 afforded a product containing only Cs^+^.

In addition, ion-exchange with a mixture of Na^+^, K^+^, Rb^+^ and Cs^+^ in a ratio of 10 : 10 : 10 : 1 yielded an exchanged compound with no Na^+^ and K^+^, Rb^+^, Cs^+^ with a ratio of 1 : 4.6 : 6.3. Thus, despite the large excess of competitive ions, the compound shows high preference for Cs^+^. The ion-exchange selectivity of this compound is probably due to its microporous framework, which excludes ions with large hydration spheres such as Na^+^ and allows the entrance of ions with limited hydration shells, such as Cs^+^. **MSIEs** containing relatively rare elements such as Ge, In, and Ga are not cost-effective; however, their study contributes to our understanding of Cs^+^ and other ion capture processes.

### 3.3. Other crystalline three-dimensional metal sulfides

There are a number of additional **MSIEs** with 3-D structures for which some ion-exchange properties were reported. Characteristic examples are open framework compounds based on T2 (*e.g.* Ge_4_S_10_^4–^) or T3 (*e.g.*In_10_S_20_^10–^) supertetrahedral units, which can exchange their extra-framework organic cations with alkali ions.^46^ Another example is K_2_Sn_2_S_5_ (KTS-2), which shows exchange capacity for Cs^+^, Sr^2+^, Pb^2+^, Cd^2+^ and Hg^2+^.^36^ However, no detailed ion-exchange studies have been performed for these materials.

In this review we focused on metal sulfides rather than selenides and tellurides, because the relatively high toxicity of selenium and tellurium makes them impractical for environmental applications. Nevertheless, it should be mentioned that metal selenides also exhibit interesting ion-exchange properties.^47^ Characteristic examples are (NH_4_)_4_In_12_Se_20_47*a* showing selective heavy metal ion sorption properties and (Cs_6_Cl)_2_Cs_5_[Ga_15_Ge_9_Se_48_]47*b* displaying both cation and anion exchange capacity.

## 4. Chalcogels with sorption properties for heavy metal ions

**Fig. 32:**
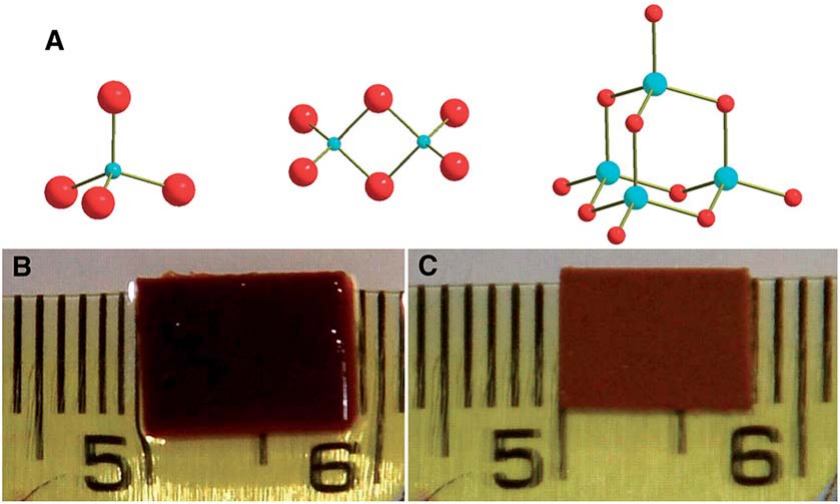
(A) Different building blocks for the formation of chalcogels. (B) Monolithic gel composed of [Ge_4_S_10_]^2–^ building blocks linked by Pt^2+^ ions before supercritical drying. (C) The chalcogel after supercritical drying. Reproduced with permission from ref. 48*a* © 2007, American Association for the Advancement of Science.

Chalcogels are gels based on all chalcogenide frameworks (Fig. 32).^48^ They are usually prepared *via* a metathesis reaction involving anionic metal chalcogenide units (Fig. 32) and linking metal ions.

These are highly porous materials combining various interesting properties, such as catalytic activity, photoluminescence, selective gas adsorption, and sorption of organic and inorganic pollutants.^48^

**Fig. 33:**
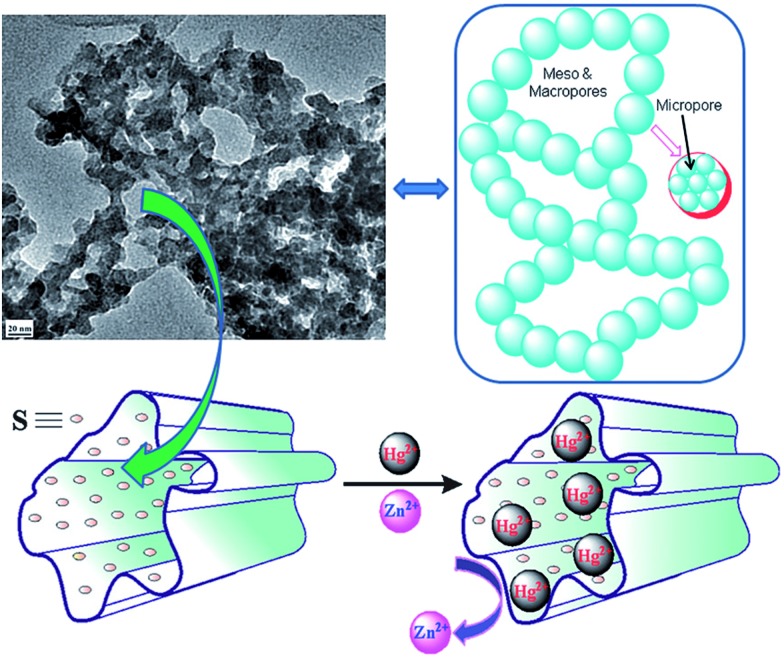
Schematic for the selective capture of soft heavy metal ions by chalcogels.

Chalcogels have proven to be excellent sorbents for Hg^2+^.48*a* Specifically, chalcogen-1 which is composed of [Ge_4_S_10_]^2–^ units linked by Pt^2+^ shows nearly 100% removal capacity for Hg^2+^ solutions. *K*_d_ values for Hg^2+^ sorption were found to be enormous (up to 10^7^ mL g^–1^), revealing the potential of chalcogels as heavy metal ion sorbents. The chalcogels show preference for the softer ions, since competitive experiments with the simultaneous presence of Hg^2+^ and Zn^2+^ indicated only Hg^2+^ absorption.48*d*Fig. 33 provides a schematic for the capture of heavy metal ions by chalcogels.

Recently, metal polysulfide chalcogels with anionic frameworks and easily exchangeable cations have been reported. Examples of such materials are KCo_6_S_21_, K–Pt–S_*x*_ and (NH_4_)_0.2_MoS_4_.^15^ They show ion-exchange capacity, as demonstrated by the complete replacement of their extra-framework cations by Cs^+^. These materials, with a combination of significant porosity, soft polysulfide ligands and highly mobile cations, appear to be particularly promising as ion-exchange materials. Thus, further studies of their ion-exchange properties would be interesting.

## 5. Engineered forms-composites of MSIEs

As described above, almost all **MSIEs** have been tested for their ion exchange properties using the so-called batch (stirring) method. Many industrial and wastewater treatment processes, however, rely on the use of continuous bed flow ion-exchange columns. A material suitable for use as a stationary phase in ion-exchange columns should combine the following characteristics: (i) high sorption capacity and rapid ion-exchange kinetics for the targeted ion, (ii) proper particle size distribution to allow continuous flow through the column and achieve the smallest possible pressure drop of the water coming through the column and (iii) sufficient mechanical strength to tolerate high water pressures. Note that for column applications, the sorbent material should generally display a particle size close to 1 to 2 mm, a size resulting from a practical compromise between limiting the pressure drop and providing adequate surface area of the sorbent for sorption of the ions.^49^**MSIEs** are usually isolated as small size crystals (≤300 μm).^13^ Thus, these materials tend to form fine suspensions in water that may either pass through the column or result in column clogging. To overcome these limitations of **MSIEs**, new approaches are essential to produce engineered forms of these materials to satisfy the requirements of the column testing. Below, we present some results on this new research direction for **MSIEs**.

### 5.1. KMS-2-alginate composite

**Fig. 34:**
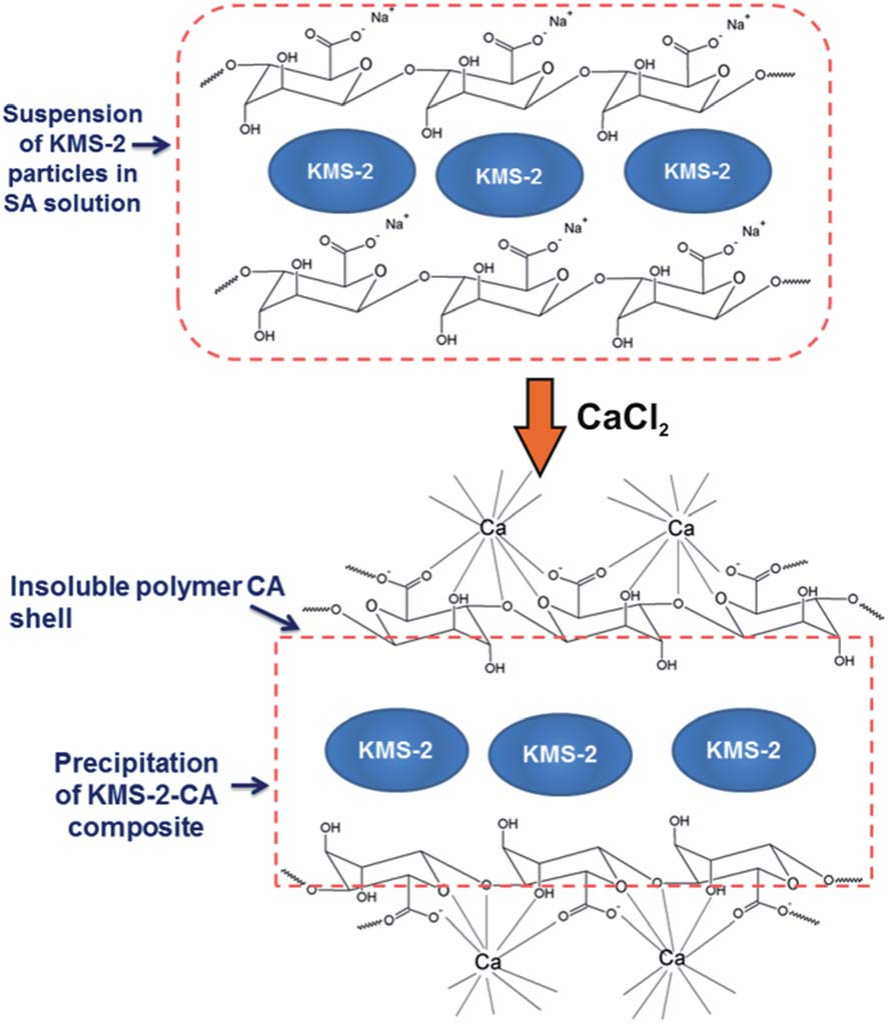
Schematic process for the isolation of the KMS-2-CA composite material.

**Fig. 35:**
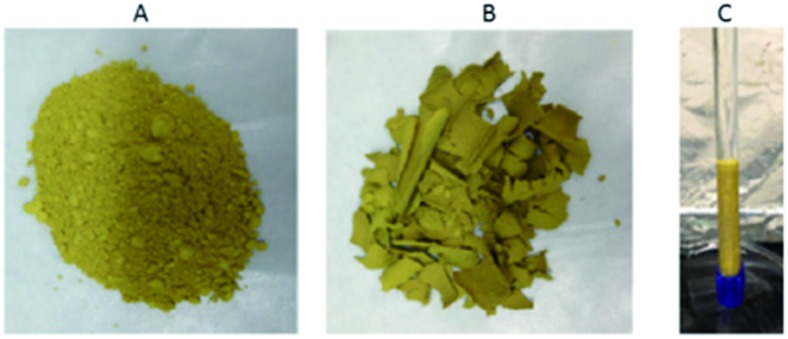
(A) Pristine layered metal sulfide (KMS-2) with particle size ≤50 μm. (B) KMS-2-CA composite (particle size ≥ 1 mm). (C) Column made with a mixture of KMS-2/CA composite (particle size 1 to 2 mm) and sand (50 to 70 mesh).

As described above, KMS-2 shows excellent batch ion-exchange properties; however, the small particle sizes (≤50 μm) preclude its use in ion-exchange columns. The alginate encapsulation method is a common way to produce materials with particles of specific shape and of suitable size for column applications.12*b* This method involves (a) addition of the sorbent to a water solution of sodium alginate (SA) so that the sorbent particles are enclosed by one or more monolayers of alginate-saturated water and (b) addition of CaCl_2_ to the SA-sorbent suspension, which results in the transformation of the alginate monolayer to a water-insoluble calcium alginate (CA) polymer encapsulating the sorbent particulates (Fig. 34).12*b* Thus, *via* the above method, paper-like KMS-2-CA composite can be prepared (Fig. 35).^50^ Note that only a small alginate content (∼4% wt) is needed to form the composite; thus, KMS-2-CA largely retains the ion-exchange properties of the pristine **MSIE** material, albeit with somewhat slower kinetics. In addition, the relatively large (mm-size) pieces of the KMS-2-CA composite are suitable for use in columns. To obtain, however, a more stable flow in the column and immobilize the KMS-2-CA particles, the composite was mixed with an inert material such as sand.

**Fig. 36:**
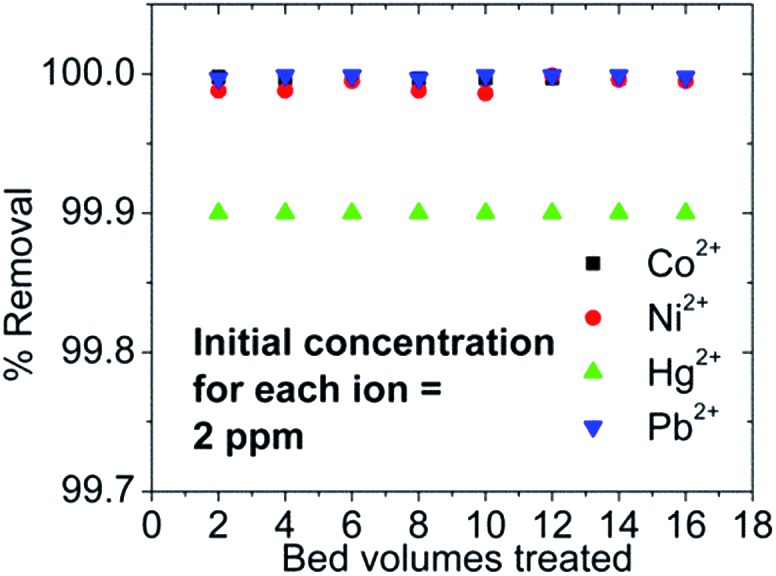
% removal of Co^2+^, Ni^2+^, Hg^2+^ and Pb^2+^*vs.* bed volume for the column ion exchange with a KMS-2-CA/sand column.

KMS-2-CA/sand (mass ratio 1 : 1) columns were tested for Ag^+^ ion-exchange. Nearly 100% Ag^+^ sorption (initial Ag concentration was 100 ppm) was observed for at least 80 bed volumes. Interestingly, the column is capable of simultaneous and almost 100% sorption of Co^2+^, Ni^2+^, Hg^2+^ and Pb^2+^ from a mixture of these ions (initial concentration ∼ 2 ppm for each of the ions) (Fig. 36). The final concentrations of all ions in the effluents were found to be well below the EPA acceptable limits.

### 5.2. KMS-1-PAN composite

**Fig. 37:**
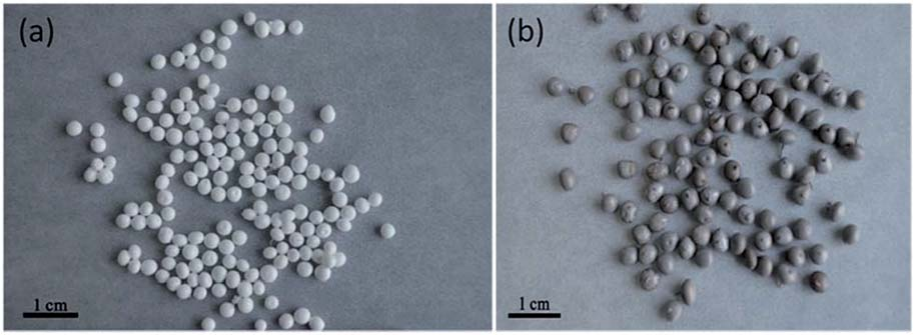
Images of (a) PAN beads, (b) KMS-1-PAN composite beads.

Polyacrylonitrile (PAN) is a binding polymer suitable for inorganic ion-exchangers. KMS-1-PAN beads (Fig. 37) were prepared by forming a suspension of KMS-1 and PAN in DMSO and adding this suspension to water, resulting in precipitation of the KMS-1-PAN composite.^51^ KMS-1-PAN composite samples with different KMS contents were prepared and tested for Cs^+^ ion-exchange with the batch method.

A sample with ∼71% wt KMS-1 showed the optimum Cs^+^ absorption. The results revealed that the composite retains the properties of the pristine KMS-1 material in a significant degree, although the Cs^+^ ion exchange is much slower with the composite. Nevertheless, the separation of the KMS-1-PAN beads from the water solution is easily accomplished.

### 5.3. Porous amorphous MSIEs

**Fig. 38:**
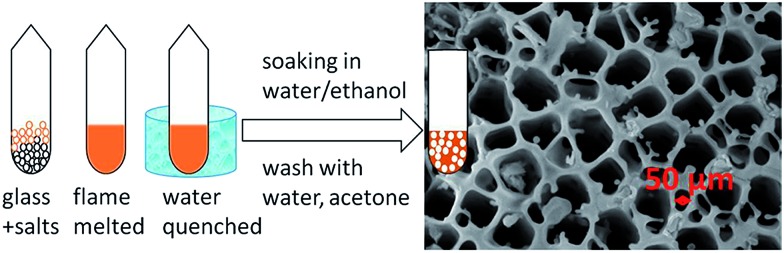
Procedure for the isolation of porous amorphous MS ion exchangers. Reproduced with permission from ref. 52 © 2015, American Chemical Society.

A different approach to produce an engineered form of **MSIE** consists of the synthesis of porous glassy materials that are melt-processable and, thus, can be made in any user defined shape and size. Porous amorphous sulfides were prepared by mixing inorganic salts with a presynthesized compound of the general formula A_2_Sn_2_SbS_6_ (A = K^+^ or Cs^+^), flame-melting the mixture, rapid quenching in water and liquid extraction of the salt (Fig. 38).^52^

The materials with the composition Na_2–*x*_K_*x*_Sn_2_SbS_6_ and Cs_2–*x*_K_*x*_Sn_2_SbS_6_ were tested for Hg^2+^, Pb^2+^ and Cd^2+^ ion-exchange. Both materials perform excellently as ion-exchangers for these soft metal ions. 99.99% Hg^2+^ removal capacities and enormous *K*_d_ values of 6.2 × 10^7^ to 7.2 × 10^8^ mL g^–1^ were obtained with these ion exchangers. In addition, significant removal capacities (70–90%) were observed for Pb^2+^ and Cd^2+^. Considering that these materials are isolated as melts that can be cut in particles of specific size and shape, they may be appropriate for column testing. Column ion-exchange studies therefore would be interesting.

## 6. Comparison between MSIEs

Some ion-exchange characteristics of representative **MSIEs** are summarized in Table 1. It can be seen that all materials show highly efficient Cs^+^ ion exchange properties, with significant sorption capacities in a relatively wide pH range, rapid sorption kinetics and moderate to excellent selectivity for Cs^+^*vs.* Na^+^.

The highest capacity for Cs^+^ is shown by KMS-2;13*i* however, the most selective Cs^+^**MSIE** is K_6_MS, which exhibits molecular sieve properties excluding ions with large hydration spheres such as Na^+^ and Ca^2+^.13*b* At the same time, one of its cavities is an exact fit for Cs^+^; thus, the *K*_d_ values for Cs^+^ exchange are high (10^3^ to 10^4^ mL g^–1^) even with a ≥10^5^-fold excess of Na^+^ or Ca^2+^.

The **MSIE** with the highest Sr^2+^ exchange capacity is KTS-3;13*i* however, the most selective sorbent is KMS-1, showing *K*_d_ ≥ 10^5^ mL g^–1^ in the presence of a ∼1900-fold excess of Na^+^.13*c* KMS-1 also showed the highest Hg^2+^ sorption capacity;13*e* however, LHMS-1 is an exceptional sorbent for Hg^2+^, even under extremely acidic conditions (pH ≤ 0).13*f* K_6_MS was also studied for its Hg^2+^ ion exchange properties, and the results also indicated the exceptional capacity and selectivity of this material to absorb Hg^2+^.^43^ KMS-113*e* and its methylammonium analogue CMS^23^ show excellent selectivity for Pb^2+^ and Cd^2+^ even in the presence of a tremendous excess of Na^+^ or Ca^2+^. CMS exhibits higher Pb^2+^ and Cd^2+^ sorption capacities but somewhat slower sorption kinetics than KMS-1. KMS-1 also exhibits high affinity for Cu^2+^ ion in the presence of Na^+^ and Ca^2+^.

KMS-113*g* and KTS-313*i* show similar UO_2_^2+^ exchange properties. Both materials exhibit very high UO_2_^2+^ removal capacities which are only slightly affected by hard ions such as H^+^, Na^+^ or Ca^2+^.

Furthermore, KMS-2 has significantly higher Ni^2+^ sorption capacity13*h* than KMS-1; however, both materials display similar and exceptional selectivity for Ni^2+^ (*K*_d_ ≥ 10^5^ mL g^–1^) in the presence of a ≥10^4^-fold excess of Na^+^.

Finally, K_6_MS is the only MS exchanger investigated for its Tl^+^ sorption properties.^43^ The first results of these investigations revealed the high Tl^+^ sorption capacity of the material and very high *K*_d_ values for Tl^+^ exchange.

## 7. Comparison of MSIE with other sorbents

At this point, it will be useful to compare the metal ion sorption properties of **MSIEs** with those of other sorbents. The unique characteristic of **MSIE** materials is their soft Lewis basic frameworks, which favor stronger interactions with soft, borderline Lewis acids and the heavier alkali and alkaline earth metal ions, in contrast to the interactions favored by oxidic materials. In addition, typical hard ions, such as H^+^, Na^+^, and Ca^2+^, which strongly interact with oxides, have relatively low or negligible effect on the ion-exchange properties of **MSIEs**. Thus, **MSIE** materials are effective for ion-exchange in the pH range of 1 to 12, whereas typical oxidic sorbents are inactive for pH ≤ 3–4.^9^,^10^ Furthermore, as discussed above, **MSIEs** achieve very high metal ion removal capacities in the presence of high excesses of Na^+^ or Ca^2+^ (and, presumably, other ions such as Mg^2+^ and Al^3+^), which in relatively high concentration cause a drastic decrease of the sorption capacity of oxides.^6^,^7^

Comparing the **MSIEs** with sulfur-functionalized materials, it can be seen that both types of materials are highly efficient and selective for the sorption of heavy metal ions. However, **MSIEs**, *e.g.* the KMS layered materials, are effective for simultaneous removal of various toxic metal ions, such as Hg^2+^, Pb^2+^, Cd^2+^,Ni^2+^, Co^2+^, and UO_2_^2+^, whereas thiol-containing mesoporous silica tends to absorb mainly Hg^2+^.^11^ Thus, **MSIEs** may find broader applications in the field of heavy metal ion remediation. The inexpensive hydrothermal synthesis of **MSIEs** is much more attractive than the multistep preparation of sulfur-functionalized materials requiring high cost organic reagents and solvents.^11^

Several studies also indicated that **MSIEs** are extremely stable in air and in both acidic (up to pH ∼ 1) and alkaline (up to pH ∼ 13 to 14) water. Their stability in water seems superior to that of silicates and aluminosilicates, which are soluble in both acidic (pH < 3) and alkaline (pH > 9) environments.^8^

A drawback of **MSIEs** is the lack of regeneration capability and reusability after being saturated with heavy metal ions. The very high capacities (up to 50% heavy metal by weight) can compensate for this non-regenerability. An exception to this rule is UO_2_^2+^-loaded and Cs^+^ loaded **MSIEs**, which can be easily converted to pristine phases; the regenerated materials can be reused for Cs^+^ and UO_2_^2+^ sorption.13d,g In some cases, **MSIEs** can often be regenerated after the Cs^+^ exchange process and reused with no loss of their exchange capacity.13*d* It is not always necessary, however, to regenerate a sorbent because this causes large amount of secondary liquid waste that needs to be stored. Solid waste occupies much less space, and if it is very stable, it can be properly buried without concern. The heavy metal ion-containing **MSIEs** can be probably considered as ultimate solid waste safe for disposal, since preliminary investigations indicated minimum leaching of heavy metals from these materials.13e,f,^28^

## 8. Conclusions and prospects

**MSIEs** represent a recently developed and growing class of ion-exchangers, which seems highly promising for environmental remediation applications. They combine various attractive features, such as potential for low cost synthesis, rapid sorption kinetics, high capacity and exceptional selectivity for toxic cations. At the same time, they do not require functionalization, since selectivity for soft ions is innate to these materials. Concerning the sorption of soft heavy metal ions, **MSIEs** outperform any other known material class. The sorption of heavy metal ions by **MSIEs** represents a textbook case of the Pearson's hard-soft-acid–base theory^53^ in action: Soft Lewis acids, such as Hg^2+^, Cd^2+^, and Pb^2+^, are preferentially absorbed by the soft basic (S^2–^-containing) framework of **MSIEs**. Waste minimization is one of the most pressing environmental issues currently facing society, and **MSIEs** by virtue of their high loading capacities could play a significant role in meeting this challenge.

Despite progress in the research on **MSIEs**, this class of ion-exchange materials remains largely unexplored. There are many known phases that may be excellent candidates as ion exchangers; however, their ion-exchange properties were overlooked in the past and were never studied in detail. These include some microporous^46^ and layered^54^ metal sulfide materials templated by organic cations, as well as various all-inorganic compounds with labile extra-framework alkali ions.^41^ Of course, exploratory synthesis may be also a fruitful source of **MSIEs**. The recent development of engineered forms and composites of **MSIEs** is an important milestone, as it forecasts good potential for many real-world applications in the field of wastewater treatment.

Finally, it would be challenging to develop metal sulfide materials that can capture toxic anionic species, such as dichromate, arsenate, and cyanate anions. A possible approach towards this challenge would be the intercalation of species into layered metal sulfide cation exchangers that will have high affinity for specific anions. Of course, the development of **MSIEs** with anion-exchange properties will open an entirely new research direction in the area of ion exchange materials. This task may require the development of cationic metal sulfides. The synthesis of such materials is feasible, as revealed by the recent reports of layered cationic metal chalcogenides.^55^ Moving forward, a wealth of research opportunities thus exist for **MSIEs**, and further progress in the understanding of these materials is anticipated in the next few years.
